# Is hunting nonintentionally selective? A test using game bird capture‐dead recoveries

**DOI:** 10.1002/ece3.9285

**Published:** 2022-09-20

**Authors:** Emilienne Grzegorczyk, Léa Bézier, Kévin Le‐Rest, Alain Caizergues, Charlotte Francesiaz, Jocelyn Champagnon, Matthieu Guillemain, Cyril Eraud

**Affiliations:** ^1^ Office Français de la Biodiversité, Conservation et Gestion Durable des Espèces Exploitées Villiers‐en‐Bois France; ^2^ Institut français de recherche pour l'exploitation de la mer Nantes France; ^3^ Office Français de la Biodiversité, Conservation et Gestion Durable des Espèces Exploitées Nantes France; ^4^ Office Français de la Biodiversité, Conservation et Gestion Durable des Espèces Exploitées Juvignac France; ^5^ Tour du Valat, Research Institute for Conservation of Mediterranean Wetlands Arles France; ^6^ Office Français de la Biodiversité, Conservation et Gestion Durable des Espèces Exploitées Arles France; ^7^ Office Français de la Biodiversité, Conservation et Gestion des Espèces à enjeux Villiers‐en‐Bois France

**Keywords:** evolution, harvest, hunting, selectivity, vulnerability, wildlife management

## Abstract

Selective hunting has various impacts that need to be considered for the conservation and management of harvested populations. The consequences of selective harvest have mostly been studied in trophy hunting and fishing, where selection of specific phenotypes is intentional. Recent studies, however, show that selection can also occur unintentionally. With at least 52 million birds harvested each year in Europe, it is particularly relevant to evaluate the selectivity of hunting on this taxon. Here, we considered 211,806 individuals belonging to 7 hunted bird species to study unintentional selectivity in harvest. Using linear mixed models, we compared morphological traits (mass, wing, and tarsus size) and body condition at the time of banding between birds that were subsequently recovered from hunting during the same year as their banding, and birds that were not recovered. We did not find any patterns showing systematic differences between recovery categories, among our model species, for the traits we studied. Moreover, when a difference existed between recovery categories, it was so small that its biological relevance can be challenged. Hunting of birds in Europe therefore does not show any form of strong selectivity on the morphological and physiological traits that we studied and should hence not lead to any change of these traits either by plastic or by evolutionary response.

## Graphical abstract

In this study, we evaluated the potential unintentional selectivity of bird hunting in Europe on 7 species. We compared morphological traits (mass, wing and tarsus size) and body condition at the time of banding between birds that were subsequently recovered from hunting and birds that were not recovered. Hunting of birds in Europe does not show any form of strong selectivity on the traits that we studied, and should hence not lead to any change of these traits either by plastic or evolutionary response.

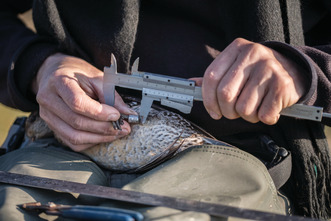

## 1 INTRODUCTION

A large number of wildlife species are harvested worldwide for commercial, recreational, or subsistence purposes (Di Minin et al., 2021; Kyle & Wilson, 2007; Ticktin, 2004). Overexploitation is one of the main threats to biodiversity and ecosystem functioning (IPBES, 2019). Assessing the consequences of harvest on the viability of species and populations is therefore a major conservation issue.

To date, the impact of harvest has mainly been addressed by assessing its consequences on population dynamics, genetic diversity, or structural/functional properties of communities (Allendorf et al., 2008; Jennings et al., 1999; Lebreton, 2005). Maintaining annual population growth rate equal or above 1 is the central objective pursued by management policies implemented to ensure the sustainability of exploitation regimes (e.g., bag limits, harvesting periods, and size limits [Lormée et al., 2020]). However, the possible evolutionary consequences have also been considered as relevant in the framework of sustainable harvesting (Allendorf & Hard, 2009). Classical examples of undesirable evolutionary outcome of harvesting include temporal changes in the frequencies of the different fur morphs in the Red Fox in response to selective harvesting by Canadian trappers (Haldane, 1942), reduction in the size of horns of bighorn sheep as a result of trophy hunting (Coltman et al., 2003; Pigeon et al., 2016), or decreasing size and age at sexual maturity observed in some fish species in which larger individuals are targeted (Law, 2000). This suggests that particular harvesting methods/regimes that target individuals according to their phenotype can impose a form of “*unnatural selection*,” sometimes in opposition to natural or sexual selection (Allendorf & Hard, 2009; Conover, 2007). However, phenotypic changes are only possible when the selective pressure induced by hunting is very high. In such conditions, the rates of phenotypic change induced by harvesting would be dramatically faster than those measured in response to other anthropogenic or natural perturbations (Darimont et al., 2009). Thus, managers should be aware that harvesting can remove genetic variability in such a way that it can seriously threaten the long‐term viability of populations (“*the Darwinian debt*,” Allendorf & Hard, 2009).

To our knowledge, the evolutionary consequences of human exploitation of wildlife species are overlooked in current sustainable exploitation strategies, which may be explained by several reasons. First, knowledge about the extent and magnitude of the evolutionary impact of harvest is scanty in most taxa. In most cases, details about the phenotypic traits potentially affected (e.g., morphology, sexual characters, and behavior) are lacking. Second, the mechanisms underlying phenotypic changes of harvested populations are still a matter of controversy, and some authors suggesting that phenotypic plasticity, not selection, is the main driver of phenotypic changes (Heffelfinger, 2018). Third, it is generally implicitly assumed that selection processes can occur if and only if harvest target a particular phenotype, intentionally (Festa‐Bianchet, 2017). However, evidence is accumulating that even nondeliberately selective harvest may differentially remove phenotypes (Leclerc et al., 2017). In this context, evaluating the occurrence of nonrandom removal of individuals is therefore the first step in assessing its possible consequences at evolutionary level (Festa‐Bianchet & Mysterud, 2018).

Numerous mechanisms can explain why nonrandom removal can result from nonconsciously selective harvesting. These may include human cognitive biases (e.g., hunters may be unconsciously tempted to remove bigger individuals) or spatiotemporal heterogeneity of the distribution of hunters respective to different phenotypes (e.g., Christensen et al., 2017). Harvested individuals may also be unintentionally selected if some intrinsic characteristics make them more vulnerable to harvest (Madden & Whiteside, 2014; Morez et al., 2000). For example, many studies suggest that bolder individuals or those moving more and over greater distances are more vulnerable to hunting or angling (Biro & Post, 2008; Ciuti et al., 2012; Madden & Whiteside, 2014; Andersen et al., 2017; see Leclerc et al., 2017 for a review). Given that phenotypic traits do not evolve in isolation (Réale et al., 2010), behavioral differences causing contrasts in vulnerability among individuals have the potential to translate into a gradual change in other heritable phenotypic characters, including morphometric traits.

In this study, we assessed whether some sort of selectivity on morphological traits or body condition may occur in migratory game bird species, even when no particular phenotype is consciously targeted by hunters. Birds are among the taxa the most exposed to harvest worldwide. Only in Europe, 10 of millions of individuals are harvested each year (Hirschfeld et al., 2019). Despite such high levels of harvest, little attention has been devoted to the study of the potential selective action of bird hunting. Existing studies suggest that hunting can be nonintentionally selective, removing weakest (waterfowl, Greenwood et al., 1986; Heitmeyer et al., 1993; Reinecke & Shaiffer, 1988) or boldest individuals (*Phasianus colchicus*, Madden & Whiteside, 2014). However, they do not allow to draw firm conclusions about the potential of harvest to imprint a form of unintentional selection at the scale of whole populations. Indeed, these studies primarily concern small geographical scales and particular hunting methods. Conversely, all hunting modes should be considered, and their consequences should be measured at relevant biological and spatial scales. This is particularly true for migratory birds, which use vast migration flyways and are therefore exposed to a wide variety of hunting modes and pressures in relation to local economic and cultural contexts. To overcome these issues, we used extensive data on morphometric traits measured as part of national banding schemes in France, whereby individuals could later be recovered anywhere in Europe. Our dataset encompassed hundreds of thousands of banded individuals belonging to seven common game species including four Anatidae, one Turdidae, one Rallidae, and one Scolopacidae. We assessed the potential selective effect of hunting by comparing morphologies (wing length, tarsus length, and body mass) and body condition (mass/wing length ratios) at banding between banded individuals, depending on whether they were later recovered by hunters (shot) or not. Under the hypothesis that hunters unintentionally may remove some phenotypes more frequently than others, we predicted differences in body mass, folded wing length, and tarsus length between birds recovered due to hunting and those not recovered, without any specific prediction regarding the direction of any potential difference. Based on several studies showing that hunting may preferentially take individuals in poor body condition in waterbirds (Greenwood et al., 1986; Heitmeyer et al., 1993; Reinecke & Shaiffer, 1988), hunted individuals should have a lower body condition index than nonrecaptured individuals in Anatidae and Rallidae.

## 2 METHODS

### 2.1 Banding data

Our study was based on morphological data of game birds banded in France between 1953 and 2020. These data include ring recoveries reported from Europe, covering several putative flyways and including individuals potentially pertaining to distinct populations. With the exception of blackbirds, some banding seasons are missing before 2000. In addition, for Anatidae and Rallidae, no banding data were available between 1978 and 2000. The details of the years covered by the study and the structure of the data are presented in Appendix S1. The dataset included morphological measures recorded at the time of banding on a total 237,890 full‐grown individuals belonging to seven game species: Mallard (*Anas platyrhynchos*), Eurasian Teal (*Anas crecca*), Tufted Duck (*Aythya fuligula*), Common Pochard (*Aythya ferina*), Common Snipe (*Gallinago gallinago*), Eurasian Coot (*Fulica atra*), and Blackbird (*Turdus merula*). Direct recoveries by hunters (see definition below) totalized 4674 events, which represents on average 2% of the banded individuals.

### 2.2 Morphological data and recovery categories

The morphological data used in the analyses included body mass, tarsus length, and folded wing length at first capture/banding (the morphology of the birds at recovery is almost never known and therefore cannot be used). As a proxy of physiological condition, we used the body condition index of Peig and Green (2010), computed from the residuals of the centered‐reduced major axis regression of mass against a measure of body size (here wing length). We had kept only full‐grown fledged individuals, banded and measured from August 1 in year n, to March 31 in year *n* + 1. In this way, we excluded most individuals that had not finished their growth and whose traits would not be representative of those they would acquire when exposed to hunting. This banding period (1 August to 31 March) encompassed the various legal hunting periods of most countries over the study period. For a given year, birds were categorized as (1) harvested, when they were recovered by hunters during the same year as banding (*direct recovery*) or (2) not recovered, when their rings were not reported during the same year or reported from hunting or other causes in a later year (*indirect recovery*). Thereafter, these two recovery categories are named “hunting recovered” and “not recovered,” respectively. Direct recoveries from other or undefined mortality causes were excluded from the analyses. Such direct recoveries from nonhunting or unknown causes amounted to <15% of the total number of direct recoveries, except for blackbird (Table 1).

**TABLE 1:** Recovery data (number of individuals) available per recovery category and species

Species	Total number of birds banded	All recovery types	All recovery types (%)	Hunting recovery	Among recovered (%)	Other recoveries	Among recovered (%)	Undetermined recoveries	Among recovered (%)
Mallard	12,940	483	3.7	400	82.8	26	5.4	57	11.8
Eurasian Teal	66,300	3296	5	3049	92.5	67	2	180	5.5
Common Snipe	15,264	541	3.5	517	95.6	4	0.7	20	3.7
Tufted Duck	4674	216	4.6	198	91.7	4	1.9	14	6.5
Common Pochard	4420	296	6.7	270	91.2	11	3.7	15	5.1
Blackbird	106,348	251	0.2	82	32.66	79	31.5	90	35.9
Eurasian Coot	1860	75	4	63	84	2	2.7	10	13.3

*Note*: This table lists all the individuals used in the selectivity models. These individuals were of known age and sex, were banded between August 1st of year n and March 31st of year *n* + 1 and recovered during this same period. Individuals with no information on their mass, wing size, and tarsal size were not taken into account in this table, only individuals with at least one measurement for one of the studied traits were kept here and for the analyses.

### 2.3 Statistical analyses

To assess whether hunters unintentionally selected individuals according to their morphological or physiological traits, for each species and each trait, we considered a full mixed linear model and used a stepwise method to find the best submodel. For each of these models, the response variable was the morphological or physiological trait at the time of banding and the explanatory variables were as follows:

#### 2.3.1 Random structure

Due to population idiosyncrasies, morphological and physiological traits may be influenced by banding location (e.g., body mass of individuals could be influenced by the resources available in the banding environment, or the use of decoys; Guillemain et al., 2015) or the fieldworker taking the morphological measurements (e.g., systematic biases; Barrett et al., 1989). To accommodate for this plausible source of variability, the models were fitted using the region of banding (NUTS 3 level) as a random factor (National structures—NUTS—Nomenclature of territorial units for statistics ‐ Eurostat, 2022). In addition, to take into account fluctuations around long‐term temporal trend, year was also considered as a random factor in all models. This random structure was the same for all species except for Eurasian Coot for all traits and Tufted Duck for tarsus where the banding region was not taken into account because, for E.Coot, for example, there were only two banding regions and 98% of individuals were banded in only one of them.

#### 2.3.2 Fixed effect

The full model considered that the traits vary according to **Sex** (male vs. female) and **Age** (first‐year vs. adult), as acknowledged by previous studies showing morphological differences between different age and sex classes (e.g.,; Fairbairn et al., 2007). The interaction between age and sex was also included in the full model (**Age × Sex**). Second, the traits may also vary in response to large‐scale changes in climate and/or land use (Guillemain, Elmberg, et al., 2010; Kaňuščák et al., 2004; Yom‐Tov, 2001; Yom‐Tov et al., 2006). The **Year** variable was considered in the full model to account for this long‐term variation. Third, the morphological and physiological characteristics would exhibit variation at shorter timescale. For example, depletion of food resources or climatic conditions is known to trigger modifications of certain plastic traits such as mass (Guillemain et al., 2005; Pravosudov & Grubb, 1997). Nonrandom mortality will also modify the proportion of different phenotypes available to hunters during the year (i.e., seasonal variation, see Haramis et al., 1986; Newcomb et al., 2016). The same also applies if migration strategies differ between the morphotypes of individuals. For all these reasons, the full model included the covariate **Julian day** (from August 1st to March 31st) too. Fourth, life history strategies and migration strategies may differ between age and sex classes. If so, patterns of the inter‐ and intra‐annual trends of the traits would differ by age and sex (Cristol et al., 1999; Guillemain et al., 2009), which has been taken into account in the full model considering four interaction terms: **Age × Year**, **Age × Julian day**, **Sex × Year**, and **Sex × Julian day**. Fifth, the intra‐annual trend considered may have changed over the period studied (1953–2020) because global changes may also have modified the intra‐annual pattern of studied traits. For example, the intra‐annual variation in body mass is expected to be less pronounced now than it was in the past, for example, because of increasing winter temperatures and available food resources. We thus considered a fifth interaction term in the full model (**Julian day × year**).

Finally, the full model also considered the core of the problematic of this paper, obviously, the differences in the morphological traits according to the information available related with hunting fate, that is, recovery versus nonrecovery. The full model thus integrated the “**Recovery**” binary variable, as well as interactions with year (**Recovery × Year**) and Julian day (Recovery **×** Julian day) to account for possible changes in hunting effect within and between years. Moreover, we also tested the interaction of recovery with age and gender (**Recovery × Age** and **Recovery × Sex**).

This full model was fitted using the lme4 package and the lmer function (Bates et al., 2015). An automatic backward elimination of fixed effects has been performed on this general model using the *step* function of the LmerTest package (Kuznetsova et al., 2017). The p‐values for the fixed effects were calculated from the F‐test based on the Sattethwaite approximation, and the significance level for the fixed part elimination was α = 0.05. During the stepwise procedure, the model parameters were estimated in maximum likelihood. Once the best set of fixed effects was found, model parameters were then better estimated by restricted maximum likelihood (Zuur et al., 2009).

In all models, intra‐ and interannual variations were modeled as a function of cubic polynomials of covariates *Year* and Julian *day* in order to provide flexibility in the form of the relationships between dependent variables and covariates, since nonlinear relationships were expected (see for example Tamisier et al., 1995 for nonlinear change in duck body mass throughout the winter).

Before analysis, data were checked for possible aberrant measurements (outliers) as our modeling approach was sensitive to mean values. To do this, the modified *Z*‐score method was used and the very conservative value of 5 × MAD (median absolute deviation) was considered as a threshold (Kannan et al., 2015). By grouping the 7 species, this represented **0.12%–0.84%** of data, depending on the trait considered.

## 3 RESULTS

As expected, we observed sexual dimorphism for traits such as mass and folded wing size in several of our species, especially in Anatidae. In teal, for example, females had slightly smaller wings (about 3 mm less) and were thinner (about 16 g less). On the contrary, in snipe, there was no difference between males and females for morphological traits such as folded wing size or tarsus. In Anatidae, our models also allowed us to observe the known pattern of seasonal variation in mass during autumn and winter.

While effects retained during the automatic backward elimination process, and thus the final model, varied between species, we observed some similarities in the structure of the final models. In all step procedures, age, sex, year, and Julian day effects were retained. Part of the trait variance was also explained by the interaction between year and Julian day in all species/trait combinations except for Mallard for folded wing and E.Coot for body condition index. Other interactions between the variables age, sex, year, and Julian day varied between the final models. All final models with the estimate, standard error, confidence interval, and p‐value for each effect are detailed in Appendix S2.

Among the 26 comparisons done (7 species times up to 4 traits), in 7 cases, the final model included a significant recovery effect (alone or in interaction): wing and body condition of Teal; mass and body condition of Eurasian Coot; mass of Common Pochard; mass and body condition of Common Snipe and wing of Blackbird (see Table 2). In two cases, the recovery effect was retained in the final model (alone or in combination) but was not significant: Teal for mass and Common Pochard for tarsus. It was interesting to note that the tarsus was the only trait for which recovery effect was never retained or significant in any of the final models.

**TABLE 2:** Estimates and standard errors of the best model for each species and all studied traits (tl: Tarsus length; fwl: Folded wing length; bm: Body mass; bc: Body condition)

	Eurasian teal				Mallard				Common pochard			
	Tl	Fwl	Bm	Bc	Tl	Fwl	Bm	Bc	Tl	Fwl	Bm	Bc
Intercept	26.92 (4.43)	183.72 (0.34)	291.03 (3.88)	305.74 (4.23)	35.04 (3.57)	270.90 (0.74)	1054.41 (15.14)	113.20 (16.94)	37.99 (1.38)	212.82 (0.76)	881.92 (32.46)	944.42 (49.23)
Recovery (hunting recoveries)		−0.11 (0.04)	−0.13 (0.34)	0.02 (0.45)					−0.69 (0.88)		−8.64 (2.75)	
Sex × recovery												
Age × recovery												
Julian_day^1 × recovery				−309.98 (124.31)								
Julian_day^2 × recovery				−297.71 (116.38)								
Julian_day^3 × recovery				−321.85 (106.82)								
Year^1 × recovery		−40.22 (10.59)	−165.78 (89.38)	71.45 (113.95)					100.66 (120.83)			
Year^2 × recovery		−12.77 (10.65)	163.80 (86.14)	322.44 (105.01)					−85.11 (85.40)			
Year^3 × recovery		−10.74 (10.19)	−88.96 (87.41)	40.01 (100.80)					39.25 (31.58)			

	Tufted duck				Eurasian coot			Common snipe				Blackbird		
	Tl	Fwl	Bm	Bc	Fwl	Bm	Bc	Tl	Fwl	Bm	Bc	Fwl	Bm	Bc
Intercept	48.29 (4.53)	204.10 (0.48)	756.10 (15.33)	743.03 (18.98)	209.69 (0.39)	625.33 (10.94)	646.60 (10.91)	34.07 (0.14)	136.68 (0.28)	103.69 (0.61)	103.24 (1.05)	127.17 (0.24)	91.55 (0.30)	93.25 (0.30)
Recovery (hunting recoveries)						−5.30 (6.00)	−9.72 (6.37)			−0.53 (0.24)	−0.88 (0.31)	0.45 (0.19)		
Sex × recovery										−0.46 (0.23)				
Age × recovery						−13.08 (5.94)	−15.43 (6.33)							
Julian_day^1 × recovery														
Julian_day^2 × recovery														
Julian_day^3 × recovery														
Year^1 × recovery														
Year^2 × recovery														
Year^3 × recovery														

*Note*: For more readability only intercept and effect of the recovery, alone or interaction with other variables, are given, the set of all estimates and confidence intervals for the final models are given in Appendix S2. when some variables or interactions are not kept in the final model during the selection the box is shaded.

**FIGURE 1:**
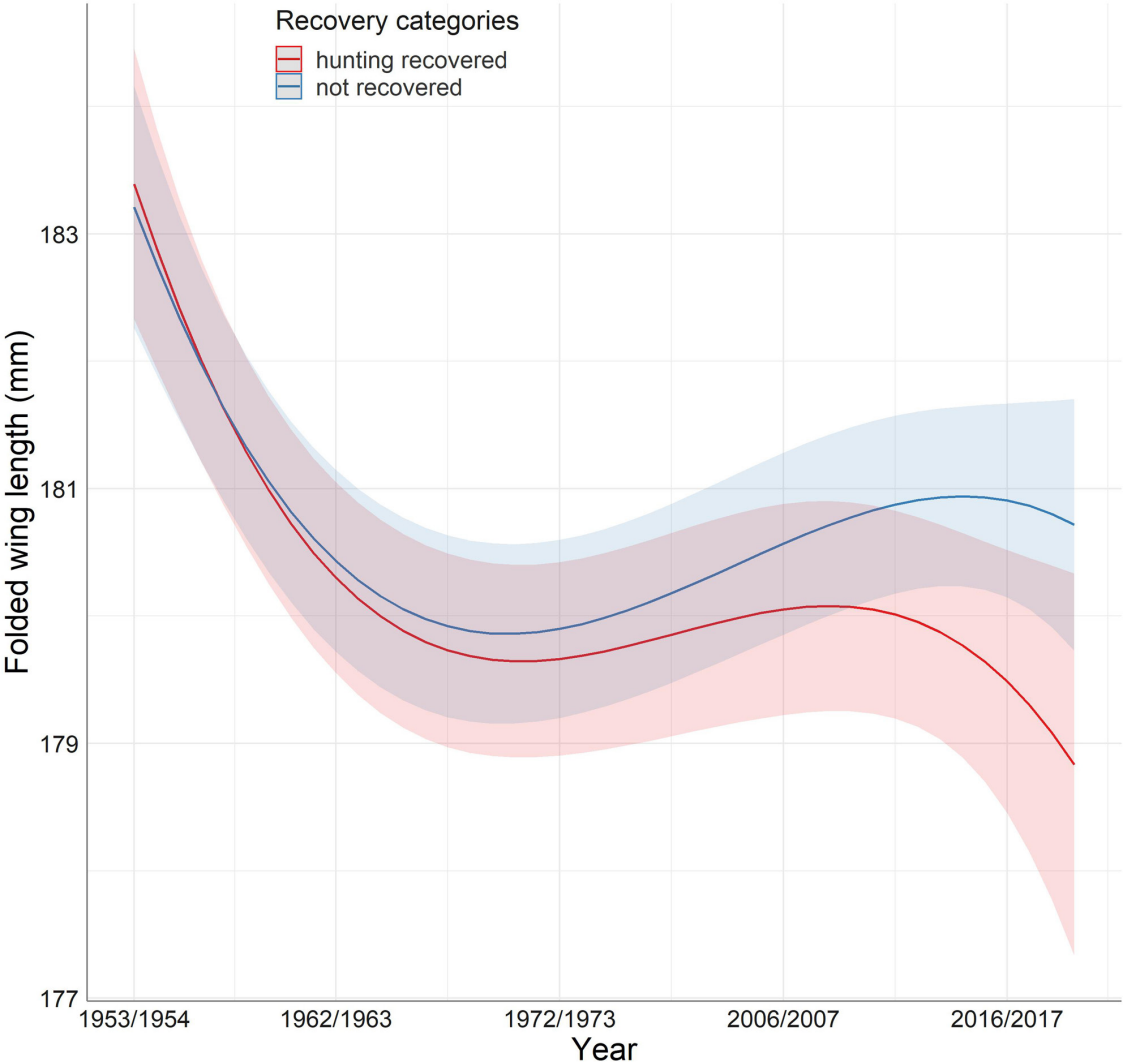
Predicted values of wing length as a function of year and recovery category in Eurasian teal (*Anas crecca*).

**FIGURE 2:**
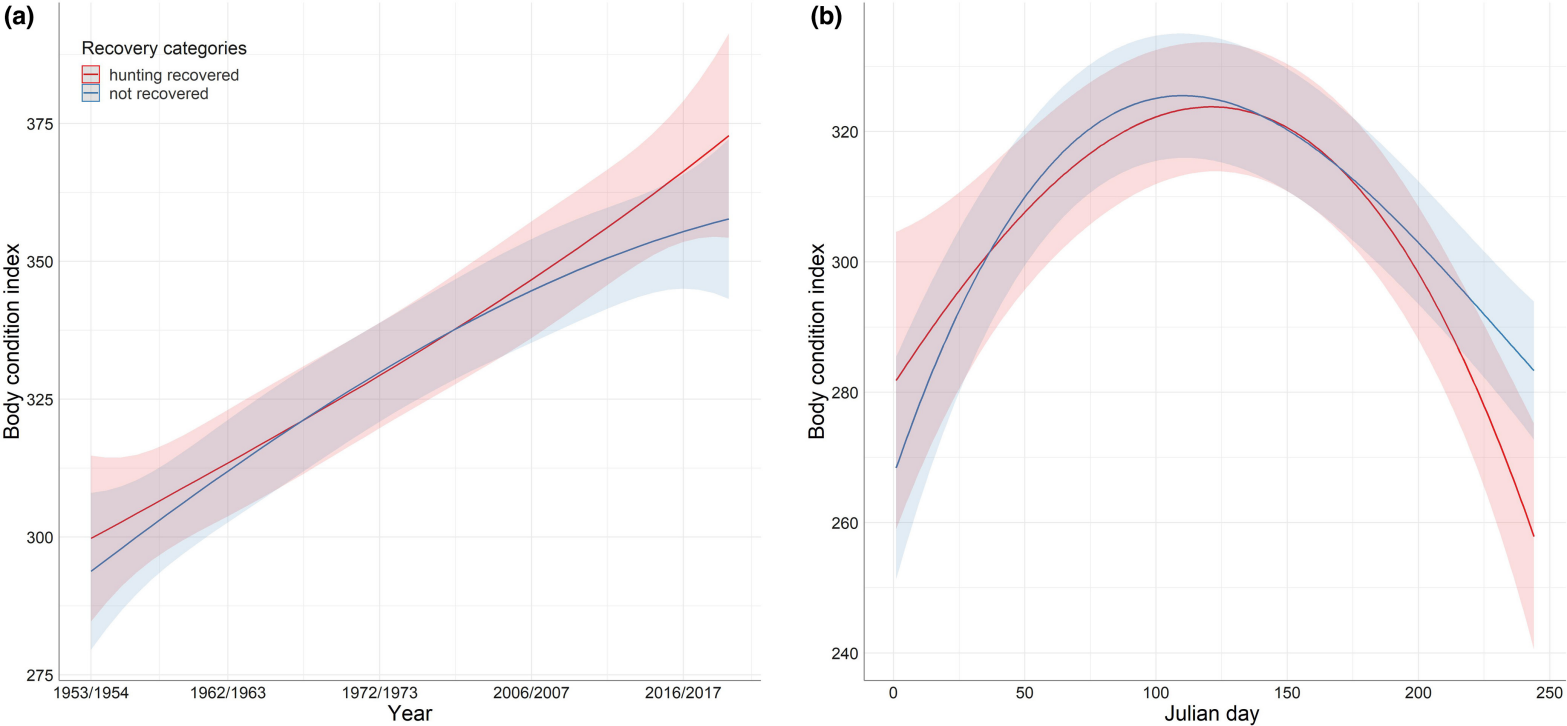
Predicted values of body condition index as a function of recovery category and year (a) or Julian day (b) in Eurasian teal (*Anas crecca*).

Even if some final models supported the hypotheses of a possible effect of hunting recovery on the trait variations, the difference between hunting recovered and unrecovered individual was low (see Table 2). In coots, the difference in mass and condition index between hunting recovery and nonrecovered coots differed according to age. In adults, birds recovered from hunting were leaner and in poorer condition than nonrecovered, but only about 37 g less (i.e., 6% of the average mass) and 50 points less (i.e., 8% of the average condition index). For juveniles, although the difference between the two categories was much smaller, the individuals recovered from hunting were heavier and in better condition, 16 g heavier (i.e., 3% of the average mass), and with a condition index 11 points higher (i.e., 2% of the average condition index). In Snipe, individuals recovered from hunting had a lower condition index than the whole available population (i.e., hunting‐recovered + nonrecovered), 0.88 (CI = −1.48 to −0.28), but this difference represented <1% of the mean value of the condition index. In the same species, there was also a small difference in mass between those recovered from hunting and those not recovered, the recovered ones were 2 g lighter (which represents 2% of the average mass), but this difference was only observed in the females. For the Common Pochard, the difference between recovered individuals and the population was 1% of the average mass (−8.64 g; CI = −14.03 to −3.24). In blackbirds for folded wing, even though it was significant, the difference between recovered birds and the population was even smaller as it represented <0.5% of the average wing size in this species (0.45. CI = 0.08–0.82). In Teal, Figure 1 shows estimated folded wing length of Teal across the study period for individuals that were hunting‐recovered and nonrecovered ones: Almost no difference existed between the two categories at the beginning and for most of the study period, only reaching ca. 2 mm greater in hunting‐recovered individuals at the end of the study period. In the same species, there was also such differences in body condition (Figure 2a). The body condition increased over the study period, and the recovered individuals had higher values at the beginning and at the end of the study period, while no difference appeared in the middle. The difference was greater at the end of the study period with birds recovered having a body condition index16 units higher than the nonrecovered birds. In this species, the difference in body condition index between recovered and nonrecovered birds also varied within the same year (Figure 2b). At the beginning, recovered individuals had a higher body condition index of about 282 compared with 269 for the nonrecovered individuals. But this difference diminished during the year and reversed at the end of the year with this time recovered individuals having a lower body condition index, about 258 compared with 284 for the nonrecovered individuals. In the few situations where statistical differences were recorded between hunting‐recovered and non‐recovered Teal, such differences were small and restricted to only a part of the dataset.

## 4 DISCUSSION

Our results do not document strong selectivity of hunting on morphological traits and body condition index of the game bird species studied. There was no trait for which final models systematically included a recovery effect. In the majority of the cases, the recovery variable was eliminated during the stepwise process. In the seven cases where the final models included an effect of recovery type, the differences between the hunting‐recovered individuals and the others were generally small, less than 1 mm difference for folded wing length in Teal, for example. The difference between the two categories of birds was slightly greater in the case of inter‐ and intra‐annual variation of body condition in Teal (Figure 2), for some parts of the year or the study period, but even in such cases, it is relevant to note that the confidence intervals of the two categories were largely overlapping, which still question the existence of a genuine marked difference between shot birds and the others.

The model selection process used here supports the list of explanatory variables and factors considered, since a large proportion of these variables were retained during the stepwise process. Moreover, average values estimated were in agreement with the literature (Del Hoyo et al., 2010). Usual differences between age or sex classes, or inter‐ and intra‐annual variations of some traits already known from the literature, such as body mass in Anatidae (Guillemain et al., 2005) were again recorded here. It is interesting to note that body mass of Anatidae increased over the study period (see Appendix S2), even though in some species such as Tufted Duck, it decreased again at the end of the study period (however, this decrease was generally accompanied by an increase in the confidence interval, which could suggest that it was simply due to a reduced number of banded individuals toward the end of the study period). In these same species, folded wing length has decreased at the same time.

Several studies show a form of selection on the traits we are studying: Morphological traits play a role in flight performance and thus in the ability to escape predators (Gosler et al., 1995; Kullberg et al., 2000; Macleod et al., 2005). However, given the very small differences in traits that we observed between hunting‐recovered and nonrecovered individuals, any fitness difference between individuals differing by less than a gram of body mass or at most 1% of the average weight is really doubtful.

Similarly, there does not appear to be any inter‐ or intra‐annual evolution of selectivity, since when the models that comprise such effects are among the best ones, the graphs of predicted values showed that the confidence intervals of recovered and nonrecovered birds almost systematically overlapped. The full set of graphs representing the interaction of recovery with Year or Julian day are available in Appendix S2.

### 4.1 Is hunting biased toward individuals of poorer quality?

So far, most studies have considered that hunters should, as natural predators do (see Hudson et al., 1992), remove poor quality individuals preferentially, especially when no particular phenotype is a priori targeted, because these individuals are more exposed to harvest (e.g., closer leak flight). Differential harvest of poor individuals should also be advised in the context of harvest management, given that a greater harvest of poorer individuals is a prerequisite toward compensatory harvest mortality (Lindberg et al., 2013) although compensatory mortality can also occur when harvesting is random if the density dependence is strong. Therefore, most previous studies have tested that hunting tends to remove poor individuals through the “lens” of body condition indexes including those employed in our studies (Christensen, 2001; Dufour et al., 1993; Greenwood et al., 1986; Hepp et al., 1986; Szymanski et al., 2013). Overall, our study does not support this formerly accepted view. This difference in results could be partly explained by the fact that some of these studies focused on the impact of certain hunting methods such as the use of decoys or hunting on wet fields (Greenwood et al., 1986; Szymanski et al., 2013), or are restricted to small geographical areas / time windows. Our study is placed in a much larger framework and sought to evaluate the selectivity of hunting in general. Indeed, by studying selectivity of hunting on a panel of relatively different migratory species, spanning entire flyways, our results likely provide a more general view over this question, hence being relevant for the management of populations and the study of possible evolutionary consequences. However, our study alone does not allow to rule out the fact that hunting can be selective under certain conditions (particular hunting methods, strong pressure).

### 4.2 Heterogeneous hunting pressure hypothesis

Another hypothesis that could explain how hunters may nonrandomly remove phenotypes relies on the possibility that hunters themselves and/or game bird phenotypes are not evenly distributed over space and time. Such “Heterogeneous Hunting Pressure” hypothesis (HPP) can explain the high frequency of unexpected migration azimuths in birds: for example, Common pochard females that migrate farther South than males experience higher predation pressure and display lower survival than males (Carbone & Owen, 1995), and in western France, sedentary and migrant Common pochards experience differential hunting mortality (see Gourlay‐Larour et al., 2014). A greater hunting pressure at lower latitudes could therefore gradually result in the promotion of unexpected directions in migration routes, such as those observed toward the North or the East during winter in diving ducks (Tableau et al. in press, see also Briedis & Bauer, 2018; Sabal et al., 2021). The HPP hypothesis could also explain unintentional selection of morphotypes. For instance, because hunting pressure is expected to increase toward the south in Europe (Hirschfeld et al., 2019), long‐distance (long‐winged) migrants (Blem, 1975; Bowlin, 2007; Hahn et al., 2016) that end up farther South than short‐distance (short‐winged) individuals should be more exposed to hunting and hence dominate the hunting bag. In our study, we considered spatiotemporal variables in our models (see materials and methods), especially because some morphological variables such as body mass show a very strong seasonal variability (Guillemain et al., 2005; Pravosudov & Grubb, 1997; Tamisier et al., 1995). By taking such variables into account, we were able to detect a slight temporal variation in the difference in traits between shot and unrecovered individuals in some cases (see graphs in Appendix S2). However, taking such spatiotemporal variables into account also prevented us, in some cases, from detecting hunting selectivity that would be due to the interaction between migration phenology and variation of the hunting pressure in space and time. If, for example, the smallest birds are the most recovered because they are more present at the beginning of the season and hunting pressure is also stronger at this time, including Julian day in the model will absorb this information. Accounting for the interaction between banding Julian day/years and recovery does not always solve this problem. In order to better account for this assumption, future analyses of hunting selectivity should attempt to explicitly take hunting pressure into account in models. However, such an exercise will be very difficult because hunting pressure depends on many factors, and such information is often difficult (when not impossible) to access, especially at flyway scale.

### 4.3 Representativity of the data

One of the major requirements in our analyses was that individuals captured for banding should be representative of the population, and in particular of individuals available for hunting. Many studies have shown there may be a bias for age, sex, size, body condition, personality, or molt in the capture of wild birds (Davis, 2005; DomèNech & Senar, 1997; Dufour & Weatherhead, 1991; Insley & Etheridge, 1997; Senar et al., 1999; Stuber et al., 2013). The biases encountered differ in particular according to the mode of capture: mist nets, clap‐net, cage, cannon nets (Davis, 2005; DomèNech & Senar, 1997; Insley & Etheridge, 1997) but also the use of callers, food or bird song to attract birds (Borras & Senar, 1986; Dufour & Weatherhead, 1991; Figuerola & Gustamante, 1995; Greenwood et al., 1986). The use of decoys to attract birds would, for example, encourage the capture of birds in poor body condition (Dufour & Weatherhead, 1991) even if this phenomenon does not seem to be systematically observed, as demonstrated by a study on two species of Sparrowhawks (Gorney et al., 1999). The fact that our dataset combines several capture methods such as mist nets, baited dabbling duck funnel traps, and cages reduces the amount of bias associated with each of these methods. Moreover banding data provided us with evidence for individual heterogeneity in survival in a number of species including many of those studied here (see Guillemain et al., 2014; Schatz, 2021). This finding suggests that both poor and good quality individuals may well be present in our banding samples, instead of banding yielding datasets biased toward some categories of individuals only. We cannot completely rule out that our data do not entirely reflect the whole range or magnitude of different phenotypes present in the population and available for hunting; however, the range of methods, the volume of birds considered, and the extended duration spanned by our datasets (several decades) make this an unlikely problem. Furthermore, it seems very unlikely that banding would select traits toward a precisely opposite direction to that potentially selected by hunting.

### 4.4 Low reporting rate may reduce statistical power

By relying on banding data to study hunting selectivity, one only has access to individuals that were shot, then recovered, and finally reported by hunters. However, it is known that some of the birds shot during the hunt are not found by the hunters, forming the “crippling loss.” In north American waterfowl, the crippling loss would vary between 20% and 40% of the birds shot (Norton & Thomas, 1994). We cannot exclude the fact that morphology is related to crippling loss, and that, for example, larger individuals fall further away and are less easily found. Moreover, even if the banded bird is found after being shot, this information is not always transmitted to the banding center by the hunters. A North American study of mallard showed band reporting probabilities to be between 0.50 and 0.81 depending on the flyway and region (Boomer et al., 2013). In our study, some of the birds classified as nonrecovered could therefore be shot birds that did not get found or reported. Such a misclassification of birds could have partly limited our ability to detect hunting selectivity on certain morphological traits. One could also think that the return of the ring is conditioned by the morphology of the bird, but as there are no regulations on the size of killed birds, nor any particular prestige in taking large individuals, such hypothesis seems very unlikely.

### 4.5 Populations may become homogenous due to the long‐standing effects

All species included in the current analyses have been quarry species for a long time. Thus, one cannot exclude that part of the variability that once allowed the expression of the full spectrum of personalities/life histories/morphologies in populations has now disappeared in response to a longstanding selective pressure exerted by harvest. If true, populations should be very homogenous not only regarding morphological traits, but also in terms of behavioral or biodemographic characteristics. Yet as already mentioned above, earlier demographic studies strongly suggests that many of our model species still display a large spectrum of demographic and behavioral traits (Gourlay‐Larour et al., 2014; Guillemain, Devineau, et al., 2010; Schatz, 2021), so again the lack of evidence for selectivity of hunting toward some morphological traits seems a genuine phenomenon.

## 5 CONCLUSION AND PERSPECTIVES

While deliberate selection of trophy ungulates or fish is a well‐known phenomenon, and sometimes even a part of harvested population management procedures, our analysis does not provide any support for unintentional selection of morphological traits during bird hunting. This is opposite to what former studies recorded, but the fact that we used a large panel of biological models over a 70 years period during which studied populations likely experienced variable environmental conditions, changing hunting modes and pressure, and hence contrasted population dynamics (increase, stability and decline) provides a strong generality to our results. Albeit we did not find strong selectivity on morphological traits, many studies show that harvest and/or natural predation nonrandomly remove personality types (see above). If a link exists between personalities and the relative contribution of different individuals to population dynamics, then such criteria may be used to promote some forms of harvest over other ones, in order to buffer the effect of exploitation at population scale. Thus, rather than simply using morphology parameters to assess population heterogeneity and (un)intentional selection by hunters, we advocate that future studies should more precisely evaluate the diversity of personalities in game populations. When sufficient heterogeneity of personalities is found, further work should aim a developing targeted management measures in order to protect those personalities which are associated with a greater contribution to population dynamics, and focus harvest toward the least contributing individuals.

## AUTHOR CONTRIBUTIONS

**Emilienne Grzegorczyk:** Conceptualization (equal); data curation (equal); formal analysis (lead); investigation (equal); methodology (equal); software (equal); validation (equal); writing – original draft (lead); writing – review and editing (lead). **Léa Bezier:** Conceptualization (equal); data curation (lead); formal analysis (lead); investigation (equal); methodology (equal); software (equal); validation (equal); writing – original draft (equal). **Kévin Le‐Rest:** Conceptualization (equal); data curation (equal); formal analysis (equal); investigation (equal); methodology (equal); software (equal); validation (equal); writing – original draft (equal); writing – review and editing (lead). **Alain Caizergues:** Conceptualization (equal); data curation (supporting); formal analysis (equal); investigation (equal); methodology (equal); validation (equal); writing – original draft (equal); writing – review and editing (equal). **Charlotte Francesiaz:** Conceptualization (equal); data curation (supporting); formal analysis (equal); investigation (equal); methodology (equal); validation (equal); writing – original draft (equal); writing – review and editing (equal). **Jocelyn Champagnon:** Conceptualization (equal); formal analysis (supporting); investigation (equal); methodology (equal); resources (equal); validation (equal); writing – original draft (equal); writing – review and editing (equal). **Matthieu Guillemain:** Conceptualization (equal); data curation (supporting); formal analysis (equal); investigation (equal); methodology (equal); supervision (supporting); validation (equal); writing – original draft (equal); writing – review and editing (equal). **Cyril Eraud:** Conceptualization (equal); data curation (supporting); formal analysis (equal); investigation (equal); methodology (equal); software (supporting); supervision (lead); validation (equal); writing – original draft (equal); writing – review and editing (equal).

## CONFLICT OF INTEREST

All authors have no conflict of interest to declare.

## Supporting information


Appendix S1

Appendix S2

## Data Availability

All data used in this article have been archived in Dryad: Grzegorczyk et al. (2022), Banding and recovery data on several game bird species to study hunting selectivity, Dryad, Dataset, https://doi.org/10.5061/dryad.8w9ghx3qh.

## References

[ece39285-bib-0001] Allendorf, F. W. , England, P. R. , Luikart, G. , Ritchie, P. A. , & Ryman, N. (2008). Genetic effects of harvest on wild animal populations. Trends in Ecology & Evolution, 23(6), 327–337. 10.1016/j.tree.2008.02.008 18439706

[ece39285-bib-0002] Allendorf, F. W. , & Hard, J. J. (2009). Human‐induced evolution caused by unnatural selection through harvest of wild animals. Proceedings of the National Academy of Sciences of the United States of America, 106(Suppl 1), 9987–9994. 10.1073/pnas.0901069106 19528656 PMC2702803

[ece39285-bib-0003] Andersen, K. H. , Marty, L. , & Arlinghaus, R. (2017). Evolution of boldness and life history in response to selective harvesting. Canadian Journal of Fisheries and Aquatic Sciences, 75, 271–281. 10.1139/cjfas-2016-0350

[ece39285-bib-0004] Barrett, R. T. , Peterz, M. , Furness, R. W. , & Durinck, J. (1989). The variability of biometric measurements. Ringing & Migration, 10(1), 13–16. 10.1080/03078698.1989.9676001

[ece39285-bib-0081] Bates, D. , Mächler, M. , Bolker, B. , & Walker, S. (2015). Fitting linear mixed‐effects models using Lme4. Journal of Statistical Software, 67 (October), 1–48. 10.18637/jss.v067.i01

[ece39285-bib-0005] Biro, P. A. , & Post, J. R. (2008). Rapid depletion of genotypes with fast growth and bold personality traits from harvested fish populations. Proceedings of the National Academy of Sciences of the United States of America, 105, 2919–2922. 10.1073/pnas.0708159105 18299567 PMC2268560

[ece39285-bib-0006] Blem, C. R. (1975). Geographic variation in wing‐loading of the house sparrow. The Wilson Bulletin, 87(4), 543–549.

[ece39285-bib-0007] Boomer, G. S. , Zimmerman, G. S. , Zimpfer, N. L. , Garrettson, P. R. , Koneff, M. D. , Sanders, T. A. , Magruder, K. D. , & Royle, J. A. (2013). Band reporting probabilities for mallards recovered in the United States and Canada. The Journal of Wildlife Management, 77(5), 1059–1066. 10.1002/jwmg.570

[ece39285-bib-0008] Borras, A. , & Senar, J. C. (1986). Sex, age and condition bias of decoy‐trapped Citril finches (*Serinus citrinella*). Miscel·lània Zoològica, 403–406.

[ece39285-bib-0009] Bowlin, M. S. (2007). Sex, wingtip shape, and wing‐loading predict arrival date at a stopover site in the Swainson's thrush (*Catharus ustulatus*). The Auk, 124(4), 1388–1396. 10.1093/auk/124.4.1388

[ece39285-bib-0010] Briedis, M. , & Bauer, S. (2018). Migratory connectivity in the context of differential migration. Biology Letters, 14(12), 20180679. 10.1098/rsbl.2018.0679 30958254 PMC6303517

[ece39285-bib-0011] Carbone, C. , & Owen, M. (1995). Differential migration of the sexes of pochard *Aythya ferina*: Results from a European survey. Wildfowl Journal, 46(46), 99–108.

[ece39285-bib-0012] Christensen, T. K. (2001). Effects of duckling body condition on hunting vulnerability in juvenile and immature common eiders Somateria mollissima’. Wildlife Biology, 7(3), 97–104. 10.2981/wlb.2001.013

[ece39285-bib-0013] Christensen, T. K. , Fox, A. D. , Sunde, P. , Hounisen, J. P. , & Andersen, L. W. (2017). Seasonal variation in the sex and age composition of the woodcock bag in Denmark. European Journal of Wildlife Research, 63(3), 52. 10.1007/s10344-017-1114-5

[ece39285-bib-0014] Ciuti, S. , Muhly, T. B. , Paton, D. G. , McDevitt, A. , Musiani, M. , & Boyce, M. S. (2012). Human selection of elk behavioural traits in a landscape of fear. Proceedings of the Royal Society B: Biological Sciences, 279(1746), 4407–4416. 10.1098/rspb.2012.1483 PMC3479801 22951744

[ece39285-bib-0015] Coltman, D. W. , O'Donoghue, P. , Jorgenson, J. T. , Hogg, J. T. , Strobeck, C. , & Festa‐Bianchet, M. (2003). Undesirable evolutionary consequences of trophy hunting. Nature, 426(6967), 655–658. 10.1038/nature02177 14668862

[ece39285-bib-0016] Conover, D. O. (2007). Nets versus nature. Nature, 450(7167), 179–180. 10.1038/450179a 17994077

[ece39285-bib-0017] Cristol, D. A. , Baker, M. B. , & Carbone, C. (1999). Differential migration revisited. In V. Nolan , E. D. Ketterson , & C. F. Thompson (Eds.), Current ornithology (pp. 33–88). Springer US. 10.1007/978-1-4757-4901-4_2

[ece39285-bib-0018] Darimont, C. T. , Carlson, S. M. , Kinnison, M. T. , Paquet, P. C. , Reimchen, T. E. , & Wilmers, C. C. (2009). Human predators outpace other agents of trait change in the wild. Proceedings of the National Academy of Sciences of the United States of America, 106(3), 952–954. 10.1073/pnas.0809235106 19139415 PMC2630061

[ece39285-bib-0019] Davis, A. K. (2005). A comparison of age, size, and health of house finches captured with two trapping methods. Journal of Field Ornithology, 76(4), 339–344. 10.1648/0273-8570-76.4.339

[ece39285-bib-0020] Del Hoyo, J. , Elliott, A. , Sargatal, J. , & Christie, D. A. (2010). Handbook of the birds of the world. Lynx Edicions. https://www.lynxeds.com/product/handbook‐of‐the‐birds‐of‐the‐world/

[ece39285-bib-0021] Di Minin, E. , Clements, H. S. , Correia, R. A. , Cortés‐Capano, G. , Fink, C. , Haukka, A. , Hausmann, A. , Kulkarni, R. , & Bradshaw, C. J. (2021). Consequences of recreational hunting for biodiversity conservation and livelihoods. One Earth, 4(2), 238–253. 10.1016/j.oneear.2021.01.014

[ece39285-bib-0022] DomèNech, J. , & Senar, J. C. (1997). Trapping methods can bias age ratio in samples of passerine populations. Bird Study, 44(3), 348–354. 10.1080/00063659709461070

[ece39285-bib-0023] Dufour, K. W. , Ankney, C. D. , & Weatherhead, P. J. (1993). Condition and vulnerability to hunting among mallards staging at Lake St. Clair, Ontario. The Journal of Wildlife Management, 57(2), 209–215. 10.2307/3809415

[ece39285-bib-0024] Dufour, K. W. , & Weatherhead, P. J. (1991). A test of the condition‐bias hypothesis using Brown‐headed cowbirds trapped during the breeding season. Canadian Journal of Zoology, 69(10), 2686–2692. 10.1139/z91-377

[ece39285-bib-0025] Fairbairn, D. J. , Blanckenhorn, W. U. , & Székely, T. (2007). Sex, size and gender roles: evolutionary studies of sexual size dimorphism. OUP Oxford.

[ece39285-bib-0026] Festa‐Bianchet, M. (2017). When does selective hunting select, how can we tell, and what should we do about it? Mammal Review, 47(1), 76–81. 10.1111/mam.12078

[ece39285-bib-0027] Festa‐Bianchet, M. , & Mysterud, A. (2018). Hunting and evolution: Theory, evidence, and unknowns. Journal of Mammalogy, 99(6), 1281–1292. 10.1093/jmammal/gyy138

[ece39285-bib-0028] Figuerola, J. , & Gustamante, L. (1995). Does use of a tape lure bias samples of curlew sandpipers captured with mist nets? (¿Afecta el Uso de Grabaciones Sonoras la Composición de las Muestras de Calidris ferruginea Capturadas con Redes Verticales?). Journal of Field Ornithology, 66(4), 497–500.

[ece39285-bib-0029] Gorney, E. , Clark, W. S. , & Yom‐Tov, Y. (1999). A test of the condition‐bias hypothesis yields different results for two species of Sparrowhawks (accipiter). The Wilson Bulletin, 111(2), 181–187.

[ece39285-bib-0030] Gosler, A. G. , Greenwood, J. J. D. , & Perrins, C. (1995). Predation risk and the cost of being fat’. Nature, 377(6550), 621–623. 10.1038/377621a0

[ece39285-bib-0031] Gourlay‐Larour, M.‐L. , Pradel, R. , Guillemain, M. , Guitton, J. S. , L'Hostis, M. , Santin‐Janin, H. , & Caizergues, A. (2014). Movement patterns in a partial migrant: A multi‐event capture‐recapture approach. PLOS One, 9(5), e96478. 10.1371/journal.pone.0096478 24802936 PMC4011787

[ece39285-bib-0032] Greenwood, H. , Clark, R. G. , & Weatherhead, P. J. (1986). Condition bias of hunter‐shot mallards (*Anas platyrhynchos*). Canadian Journal of Zoology, 64(3), 599–601. 10.1139/z86-088

[ece39285-bib-0033] Guillemain, M. , Cavallo, F. , Massez, G. , George, T. , Baudet, J. P. , Gonzalez, P. , Ducasse, V. , Caillot, E. , Lecaplain, B. , Tison, L. , & Piffeteau, N. (2015). Ringing does not appear to have an adverse effect on body mass immediately following capture in Eurasian teal Anas crecca. Wildfowl, 65(65), 64–74.

[ece39285-bib-0034] Guillemain, M. , Dehorter, O. , Johnson, A. R. , & Simon, G. (2005). A test of the wintering strategy hypothesis with teal (*Anas crecca*) ringed in the Camargue, southern France. Journal of Ornithology, 146(2), 184–187. 10.1007/s10336-005-0080-y

[ece39285-bib-0035] Guillemain, M. , Devineau, O. , Brochet, A. L. , Fuster, J. , Fritz, H. , Green, A. J. , & Gauthier‐Clerc, M. (2010). What is the spatial unit for a wintering teal Anas crecca? Weekly day roost fidelity inferred from nasal saddles in the Camargue, southern France. Wildlife Biology, 16(2), 215–220. 10.2981/09-042

[ece39285-bib-0036] Guillemain, M. , Elmberg, J. , Gauthier‐Clerc, M. , Massez, G. , Hearn, R. , Champagnon, J. , & Simon, G. (2010). Wintering French mallard and teal are heavier and in better body condition than 30 years ago: Effects of a changing environment? Ambio, 39(2), 170–180. 10.1007/s13280-010-0020-9 20653279 PMC3357686

[ece39285-bib-0037] Guillemain, M. , Hearn, R. , King, R. , Gauthier‐Clerc, M. , Simon, G. , & Caizergues, A. (2009). Differential migration of the sexes cannot be explained by the body size hypothesis in teal. Journal of Ornithology, 150(3), 685–689. 10.1007/s10336-009-0375-5

[ece39285-bib-0038] Guillemain, M. , Pradel, R. , Devineau, O. , Simon, G. , & Gauthier‐Clerc, M. (2014). Demographic heterogeneity among individuals can explain the discrepancy between capture–mark–recapture and waterfowl count results. The Condor, 116(3), 293–302. 10.1650/CONDOR-13-148.1

[ece39285-bib-0039] Hahn, S. , Korner‐Nievergelt, F. , Emmenegger, T. , Amrhein, V. , Csörgő, T. , Gursoy, A. , Ilieva, M. , Kverek, P. , Pérez‐Tris, J. , Pirrello, S. , Zehtindjiev, P. , & Salewski, V. (2016). Longer wings for faster springs – Wing length relates to spring phenology in a long‐distance migrant across its range. Ecology and Evolution, 6(1), 68–77. 10.1002/ece3.1862 26811775 PMC4716511

[ece39285-bib-0040] Haldane, J. B. S. (1942). The selective elimination of silver foxes in eastern Canada. Journal of Genetics, 44(2–3), 296–304. 10.1007/BF02982833

[ece39285-bib-0041] Haramis, G. M. , Nichols, J. D. , Pollock, K. H. , & Hines, J. E. (1986). The relationship between body mass and survival of wintering canvasbacks. The Auk, 103(3), 506–514. 10.1093/auk/103.3.506

[ece39285-bib-0042] Heffelfinger, J. R. (2018). Obstacles to evolutionary consequences of ungulate trophy hunting: Reply to Kardos et al. The Journal of Wildlife Management, 82(5), 892–895. 10.1002/jwmg.21487

[ece39285-bib-0043] Heitmeyer, M. E. , Fredrickson, L. H. , & Humburg, D. D. (1993). ‘Further evidence of biases associated with hunter‐killed mallards. The Journal of Wildlife Management, 57(4), 733–740. 10.2307/3809073

[ece39285-bib-0044] Hepp, G. R. , Blohm, R. J. , Reynolds, R. E. , Hines, J. E. , & Nichols, J. D. (1986). Physiological condition of autumn‐banded mallards and its relationship to hunting vulnerability. The Journal of Wildlife Management, 50(2), 177–183. 10.2307/3801893

[ece39285-bib-0045] Hirschfeld, A. , Attard, G. , & Scott, L. (2019). Bird hunting in Europe. British Birds, 112, 153–166.

[ece39285-bib-0046] Hudson, P. J. , Dobson, A. P. , & Newborn, D. (1992). Do parasites make prey vulnerable to predation? Red grouse and parasites. Journal of Animal Ecology, 61(3), 681–692. 10.2307/5623

[ece39285-bib-0047] Insley, H. , & Etheridge, B. (1997). Catching bias in cannon and mist netted samples of redshanks *Tringa totanus* on the inner Moray firth. Ringing & Migration, 18(1), 70–77. 10.1080/03078698.1997.9674144

[ece39285-bib-0083] Intergovernmental Science‐Policy Platform On Biodiversity And Ecosystem Services (IPBES) . (2019). Summary for Policymakers of the Global Assessment Report on Biodiversity and Ecosystem Services. Zenodo. 10.5281/ZENODO.3553579

[ece39285-bib-0049] Jennings, S. , Greenstreet, S. P. R. , & Reynolds, J. D. (1999). Structural change in an exploited fish community: A consequence of differential fishing effects on species with contrasting life histories. Journal of Animal Ecology, 68(3), 617–627. 10.1046/j.1365-2656.1999.00312.x

[ece39285-bib-0050] Kannan, K. S. , Manoj, K. , & Arumugam, S. (2015). Labeling methods for identifying outliers. International Journal of Statistics and Systems, 10(2), 231–238.

[ece39285-bib-0051] Kaňuščák, P. , Hromada, M. , Tryjanowski, P. , & Sparks, T. (2004). Does climate at different scales influence the phenology and phenotype of the river warbler *Locustella fluviatilis*? Oecologia, 141(1), 158–163. 15300486 10.1007/s00442-004-1646-8

[ece39285-bib-0052] Kullberg, C. , Jakobsson, S. , & Fransson, T. (2000). High migratory fuel loads impair predator evasion in sedge warblers. The Auk, 117(4), 1034–1038. 10.1093/auk/117.4.1034

[ece39285-bib-0053] Kuznetsova, A. , Brockhoff, P. B. , & Christensen, R. H. B. (2017). “lmerTest” package: Tests in linear mixed effects models. Journal of Statistical Software, 82(13), 1–26. 10.18637/jss.v082.i13

[ece39285-bib-0054] Kyle, C. J. , & Wilson, C. C. (2007). Mitochondrial DNA identification of game and harvested freshwater fish species. Forensic Science International, 166(1), 68–76. 10.1016/j.forsciint.2006.03.025 16690237

[ece39285-bib-0055] Law, R. (2000). Fishing, selection, and phenotypic evolution. ICES Journal of Marine Science, 57(3), 659–668. 10.1006/jmsc.2000.0731

[ece39285-bib-0056] Lebreton, J.‐D. (2005). Dynamical and statistical models for exploited populations†. Australian & New Zealand Journal of Statistics, 47(1), 49–63. 10.1111/j.1467-842X.2005.00371.x

[ece39285-bib-0057] Leclerc, M. , Zedrosser, A. , & Pelletier, F. (2017). Harvesting as a potential selective pressure on behavioural traits. Journal of Applied Ecology, 54(6), 1941–1945. 10.1111/1365-2664.12893

[ece39285-bib-0058] Lindberg, M. S. , Sedinger, J. S. , & Lebreton, J.‐D. (2013). Individual heterogeneity in black Brant survival and recruitment with implications for harvest dynamics. Ecology and Evolution, 3(12), 4045–4056. 10.1002/ece3.767 24324858 PMC3853552

[ece39285-bib-0059] Lormée, H. , Barbraud, C. , Peach, W. , Carboneras, C. , Lebreton, J. D. , Moreno‐Zarate, L. , Bacon, L. , & Eraud, C. (2020). Assessing the sustainability of harvest of the European turtle‐dove along the European western flyway. Bird Conservation International, 30(4), 506–521. 10.1017/S0959270919000479

[ece39285-bib-0060] Macleod, R. , Barnett, P. , Clark, J. A. , & Cresswell, W. (2005). ‘Body mass change strategies in blackbirds Turdus merula: The starvation–predation risk trade‐off. Journal of Animal Ecology, 74(2), 292–302. 10.1111/j.1365-2656.2005.00923.x

[ece39285-bib-0061] Madden, J. R. , & Whiteside, M. A. (2014). Selection on behavioural traits during “unselective” harvesting means that shy pheasants better survive a hunting season. Animal Behaviour, 87, 129–135. 10.1016/j.anbehav.2013.10.021

[ece39285-bib-0062] Morez, V. , Gauthier, G. , & Reed, A. (2000). Effect of body condition on vulnerability of greater snow geese to hunting and capture. The Journal of Wildlife Management, 64(3), 875–886. 10.2307/3802758

[ece39285-bib-0063] National structures ‐ NUTS ‐ Nomenclature of territorial units for statistics ‐ Eurostat (2022). https://ec.europa.eu/eurostat/web/nuts/national‐structures

[ece39285-bib-0064] Newcomb, K. C. , Davis, J. B. , Kaminski, R. M. , & Gray, M. J. (2016). Winter survival of female American black ducks in Tennessee, USA. The Condor, 118(1), 33–45. 10.1650/CONDOR-15-74.1

[ece39285-bib-0065] Norton, M. R. , & Thomas, V. G. (1994). Economic analyses of “crippling losses” of north American waterfowl and their policy implications for management. Environmental Conservation, 21(4), 347–353. 10.1017/S037689290003366X

[ece39285-bib-0066] Peig, J. , & Green, A. (2010). The paradigm of body condition: A critical reappraisal of current methods based on mass and length’. Functional Ecology, 24, 1323–1332. 10.1111/j.1365-2435.2010.01751.x

[ece39285-bib-0067] Pigeon, G. , Festa‐Bianchet, M. , Coltman, D. W. , & Pelletier, F. (2016). Intense selective hunting leads to artificial evolution in horn size. Evolutionary Applications, 9(4), 521–530. 10.1111/eva.12358 27099619 PMC4831456

[ece39285-bib-0068] Pravosudov, V. V. , & Grubb, T. C. (1997). Energy management in passerine birds during the nonbreeding season. In V. Nolan , E. D. Ketterson , & C. F. Thompson (Eds.), Current ornithology (pp. 189–234). Springer US. 10.1007/978-1-4757-9915-6_5

[ece39285-bib-0069] Réale, D. , Garant, D. , Humphries, M. M. , Bergeron, P. , Careau, V. , & Montiglio, P. O. (2010). Personality and the emergence of the pace‐of‐life syndrome concept at the population level. Philosophical Transactions of the Royal Society B: Biological Sciences, 365(1560), 4051–4063. 10.1098/rstb.2010.0208 PMC2992747 21078657

[ece39285-bib-0070] Reinecke, K. J. , & Shaiffer, C. W. (1988). A field test for differences in condition among trapped and shot mallards. The Journal of Wildlife Management, 52(2), 227–232. 10.2307/3801226

[ece39285-bib-0071] Sabal, M. C. , Boyce, M. S. , Charpentier, C. L. , Furey, N. B. , Luhring, T. M. , Martin, H. W. , Melnychuk, M. C. , Srygley, R. B. , Wagner, C. M. , Wirsing, A. J. , & Ydenberg, R. C. (2021). Predation landscapes influence migratory prey ecology and evolution. Trends in Ecology & Evolution, 36(8), 737–749. 10.1016/j.tree.2021.04.010 33994219

[ece39285-bib-0072] Schatz, C. (2021). Hétérogénéité individuelle du taux de survie des oiseaux chassables. MSc Thesis. Faculté des sciences de la vie, Université de Strasbourg. University of Strasbourg.

[ece39285-bib-0073] Senar, J. C. , Domenech, J. , & Conroy, M. J. (1999). Funnel traps capture a higher proportion of juvenile great tits parus major than automatic traps. Ringing & Migration, 19(4), 257–259. 10.1080/03078698.1999.9674189

[ece39285-bib-0074] Stuber, E. F. , Araya‐Ajoy, Y. G. , Mathot, K. J. , Mutzel, A. , Nicolaus, M. , Wijmenga, J. J. , Mueller, J. C. , & Dingemanse, N. J. (2013). Slow explorers take less risk: A problem of sampling bias in ecological studies. Behavioral Ecology, 24(5), 1092–1098. 10.1093/beheco/art035

[ece39285-bib-0075] Szymanski, M. L. , Johnson, M. A. , & Grovijahn, M. (2013). Effects of hunting pressure and collection method bias on body mass of drake mallards. The Journal of Wildlife Management, 77(2), 235–242. 10.1002/jwmg.465

[ece39285-bib-0082] Tableau, A. , Gourlay‐Larour, M.‐L. , Sorin, C. , Arcanger, J.‐F. , Guillemain, M. , Rabatel, J.‐P. , George, T. , & Caizergues, A. (in press). Movement patterns of diving ducks Aythya sp. in western Europe. Wildfowl.

[ece39285-bib-0076] Tamisier, A. , Allouche, L. , Aubry, F. , & Dehorter, O. (1995). Wintering strategies and breeding success: Hypothesis for a trade‐off in some waterfowl species. Wildfowl, 46(46), 76–88.

[ece39285-bib-0077] Ticktin, T. (2004). The ecological implications of harvesting non‐timber forest products. Journal of Applied Ecology, 41(1), 11–21. 10.1111/j.1365-2664.2004.00859.x

[ece39285-bib-0078] Yom‐Tov, Y. (2001). Global warming and body mass decline in Israeli passerine birds. Proceedings of the Royal Society of London. Series B: Biological Sciences, 268(1470), 947–952. 10.1098/rspb.2001.1592 PMC1088692 11370968

[ece39285-bib-0079] Yom‐Tov, Y. , Yom‐Tov, S. , Wright, J. , JR Thorne, C. , & Du Feu, R. (2006). Recent changes in body weight and wing length among some British passerine birds. Oikos, 112(1), 91–101. 10.1111/j.0030-1299.2006.14183.x

[ece39285-bib-0080] Zuur, A. , Ieno, E. N. , Walker, N. , Saveliev, A. A. , & Smith, G. M. (2009). Mixed effects models and extensions in ecology with R. Springer Science & Business Media

