# Supplementary material for: Is hunting nonintentionally selective? A test using game bird capture‐dead recoveries
Source: Ecol Evol. 2022 Sep 20;12(9):e9285. doi: 10.1002/ece3.9285 (PMC9486496; doi:10.1002/ece3.9285)
Supplement: Supplementary file 1 — Appendix S1 [file ECE3-12-e9285-s002.docx]

**Appendix A**

Description of the banding dataset. Individuals were of known age and sex, they were banded between August 1st of year n and March 31st of year n+1 and recovered during this same period. Individuals with no information on their mass, wing size and tarsal size were not taken into account in this table, only individuals with at least one measurement for one of the studied traits were kept here and for the analyses (hence sample size for a given species may differ among morphological traits).

| **Species** | **Total number of birds** | **Females** | **Males** | **Young** | **Adults** | **Number of banding areas** | **Study period** | **Missing season(s)** |
| --- | --- | --- | --- | --- | --- | --- | --- | --- |
| **Mallard** | 12 857 | 5 367 | 7 490 | 7 175 | 5 682 | 20 | 1953-1954 to 1977-1978  and  2001-2002 to 2019-2020 | 1974-1975 |
| **Eurasian teal** | 66 053 | 30 139 | 35 914 | 43 692 | 22 361 | 33 | 1953-1954 to 1977-1978  and  2001-2002 to 2019-2020 | 1974-1975 |
| **Common snipe** | 15 240 | 8 468 | 6 772 | 10 135 | 5 105 | 65 | 1958-1959 to 2019-2020 | 1960-1961  1962-1963 to 1978-1979  1980-1981  1982-1983  1993-1994 |
| **Tufted Duck** | 4 656 | 2 591 | 2 065 | 3 526 | 1 130 | 13 | 1955-1956 to 19771978  and  2002-2003 to 2019-2020 | 1956-1957  1974-1975 |
| **Common pochard** | 4 394 | 2 032 | 2 362 | 2 846 | 1 548 | 13 | 1954-1955 to 1977-1978  and  2003-2004 to 2019-2020 | 1974-1975  1975-1976 |
| **Blackbird** | 106 179 | 49 178 | 57 001 | 71 255 | 34 924 | 96 | 1953-1954 to 2018-2019 | None |
| **Eurasian coot** | 1 848 | 816 | 1032 | 570 | 1278 | 2 | 1954-1955 to 1977-1978  and  2008-2009 to 2014-2015 | 1974-1975 |
