# Supplementary material for: Is hunting nonintentionally selective? A test using game bird capture‐dead recoveries
Source: Ecol Evol. 2022 Sep 20;12(9):e9285. doi: 10.1002/ece3.9285 (PMC9486496; doi:10.1002/ece3.9285)
Supplement: Supplementary file 2 — Appendix S2 [file ECE3-12-e9285-s001.docx]

**Appendix B:** Final model result tables and associated graphics

The contrasts method used for all model results presented below are the following: contr.sum and contr.poly (function contrasts () in R <https://www.rdocumentation.org/packages/car/versions/3.0-12/topics/Contrasts>). Contr.sum constrains the sum of the coefficients of each category to be equal to zero. Taking the example of the specie presented (Folded wing length for Eurasian Teal), the interpretation of the Sex effect on the folded wing length is as follows: females have a folded wing 3.76 mm smaller than the population average while males have a folded wing 3.76 mm larger than the population average, the difference in folded wing length between both sexes is 7.52 mm. With the use of such contrast, for each of the models presented below the intercept estimate gives the average value of the trait in the population (both hunting recovered and non-recovered birds). Still in the Eurasian Teal, the first model estimates a mean folded wing length of 183.72 mm in the population. Cl correspond to the confidence intervals and p to the p-values.

- **Eurasian Teal**
- **Tarsus**

|  | **tarsus** | | | | | |
| --- | --- | --- | --- | --- | --- | --- |
| ***Predictors*** | ***Estimates*** | | ***std. Error*** | ***CI*** | | ***p*** |
| **(Intercept)** | 26.92 | | 4.43 | 18.23 – 35.60 | | **<0.001** |
| **Sex (female)** | -0.44 | | 0.01 | -0.47 – -0.42 | | **<0.001** |
| **Age(adult)** | 0.05 | | 0.01 | 0.03 – 0.08 | | **<0.001** |
| **Julian day ^1** | 872.01 | | 382.15 | 123.01 – 1621.00 | | **0.022** |
| **Julian day ^2** | -177.60 | | 381.38 | -925.09 – 569.88 | | 0.641 |
| **Julian day ^3** | 224.65 | | 358.14 | -477.30 – 926.60 | | 0.530 |
| **Year^1** | 581.59 | | 743.35 | -875.34 – 2038.52 | | 0.434 |
| **Year^2** | -293.99 | | 376.98 | -1032.87 – 444.88 | | 0.435 |
| **Year^3** | 65.95 | | 91.80 | -113.97 – 245.87 | | 0.472 |
| **Sex * Julian day ^1** | -2.42 | | 2.15 | -6.63 – 1.79 | | 0.260 |
| **Sex * Julian day ^2** | -3.33 | | 2.04 | -7.32 – 0.66 | | 0.101 |
| **Sex * Julian day ^3** | 4.37 | | 2.09 | 0.28 – 8.47 | | **0.036** |
| **Age * Julian day ^1** | 3.39 | | 2.20 | -0.92 – 7.70 | | 0.123 |
| **Age * Julian day ^2** | -5.78 | | 2.09 | -9.87 – -1.69 | | **0.006** |
| **Age * Julian day ^3** | -2.37 | | 2.13 | -6.54 – 1.81 | | 0.267 |
| **Julian day ^1 * Year^1** | -150504.00 | | 64085.59 | -276109.43 – -24898.56 | | **0.019** |
| **Julian day ^2 * Year^1** | 23965.34 | | 63717.72 | -100919.09 – 148849.77 | | 0.707 |
| **Julian day ^3 * Year^1** | -35374.19 | | 59982.69 | -152938.11 – 82189.72 | | 0.555 |
| **Julian day ^1 * Year^2** | 79439.64 | | 32444.72 | 15849.16 – 143030.11 | | **0.014** |
| **Julian day ^2 * Year^2** | -4402.32 | | 31974.17 | -67070.54 – 58265.90 | | 0.890 |
| **Julian day ^3 * Year^2** | 15053.15 | | 30298.48 | -44330.78 – 74437.09 | | 0.619 |
| **Julian day ^1 * Year^3** | -21039.38 | | 7961.50 | -36643.64 – -5435.13 | | **0.008** |
| **Julian day ^2 * Year^3** | -1152.37 | | 7740.61 | -16323.70 – 14018.96 | | 0.882 |
| **Julian day ^3 * Year^3** | -1871.78 | | 7399.19 | -16373.92 – 12630.36 | | 0.800 |
| **Random Effects** | | | | |  |  |
| σ^2^ | | 1.23 | | |  |  |
| τ_00_ _banding_area_ | | 0.46 | | |  |  |
| τ_00_ _year_ | | 0.02 | | |  |  |
| ICC | | 0.28 | | |  |  |
| N _banding_area_ | | 33 | | |  |  |
| N _year_ | | 18 | | |  |  |
| Observations | | 11473 | | |  |  |
| Marginal R^2^ / Conditional R^2^ | | 0.112 / 0.362 | | |  |  |

- **Folded wing length**

|  | **folded_wing** | | | | | |
| --- | --- | --- | --- | --- | --- | --- |
| ***Predictors*** | ***Estimates*** | | ***std. Error*** | ***CI*** | | ***p*** |
| **(Intercept)** | 183.72 | | 0.34 | 183.06 – 184.38 | | **<0.001** |
| **Sex (female)** | -3.76 | | 0.02 | -3.80 – -3.72 | | **<0.001** |
| **Age(adult)** | 0.98 | | 0.02 | 0.94 – 1.02 | | **<0.001** |
| **Julian day ^1** | -23.44 | | 5.84 | -34.89 – -12.00 | | **<0.001** |
| **Julian day ^2** | 5.67 | | 6.17 | -6.41 – 17.76 | | 0.358 |
| **Julian day ^3** | -54.67 | | 5.51 | -65.47 – -43.86 | | **<0.001** |
| **Year^1** | -85.63 | | 24.98 | -134.59 – -36.67 | | **0.001** |
| **Year^2** | 123.03 | | 23.52 | 76.92 – 169.13 | | **<0.001** |
| **Year^3** | -90.28 | | 20.23 | -129.94 – -50.62 | | **<0.001** |
| **Recovery (Hunting recovery)** | -0.11 | | 0.04 | -0.19 – -0.03 | | **0.009** |
| **Sex * Age** | -0.33 | | 0.02 | -0.37 – -0.29 | | **<0.001** |
| **Sex * Julian day ^1** | -7.33 | | 4.49 | -16.13 – 1.47 | | 0.102 |
| **Sex * Julian day ^2** | 19.54 | | 4.69 | 10.36 – 28.73 | | **<0.001** |
| **Sex * Julian day ^3** | 5.75 | | 4.41 | -2.90 – 14.40 | | 0.193 |
| **Sex * Year^1** | -14.98 | | 4.52 | -23.84 – -6.12 | | **0.001** |
| **Sex * Year^2** | 24.08 | | 4.58 | 15.10 – 33.07 | | **<0.001** |
| **Sex * Year^3** | -0.48 | | 4.32 | -8.95 – 7.98 | | 0.911 |
| **Age * Julian day ^1** | 5.22 | | 5.04 | -4.66 – 15.10 | | 0.301 |
| **Age * Julian day ^2** | -50.23 | | 5.19 | -60.40 – -40.05 | | **<0.001** |
| **Age * Julian day ^3** | -1.97 | | 4.83 | -11.43 – 7.49 | | 0.683 |
| **Age * Year^1** | -34.47 | | 4.88 | -44.03 – -24.91 | | **<0.001** |
| **Age * Year^2** | 1.05 | | 4.79 | -8.33 – 10.43 | | 0.826 |
| **Age * Year^3** | 2.69 | | 4.48 | -6.08 – 11.46 | | 0.548 |
| **Julian day ^1 * Year^1** | 4200.54 | | 1245.99 | 1758.45 – 6642.64 | | **0.001** |
| **Julian day ^2 * Year^1** | -4649.45 | | 1315.40 | -7227.58 – -2071.32 | | **<0.001** |
| **Julian day ^3 * Year^1** | 7125.78 | | 1145.07 | 4881.49 – 9370.07 | | **<0.001** |
| **Julian day ^1 * Year^2** | -8528.00 | | 1531.71 | -11530.09 – -5525.90 | | **<0.001** |
| **Julian day ^2 * Year^2** | 2007.89 | | 1616.95 | -1161.26 – 5177.05 | | 0.214 |
| **Julian day ^3 * Year^2** | -3105.52 | | 1408.41 | -5865.95 – -345.09 | | **0.027** |
| **Julian day ^1 * Year^3** | 1215.37 | | 1155.96 | -1050.28 – 3481.01 | | 0.293 |
| **Julian day ^2 * Year^3** | -3094.09 | | 1186.05 | -5418.71 – -769.47 | | **0.009** |
| **Julian day ^3 * Year^3** | 1950.71 | | 1093.28 | -192.08 – 4093.51 | | 0.074 |
| **Year^1 * Recovery** | -40.22 | | 10.59 | -60.97 – -19.46 | | **<0.001** |
| **Year^2 * Recovery** | -12.77 | | 10.65 | -33.63 – 8.10 | | 0.230 |
| **Year^3 * Recovery** | -10.74 | | 10.19 | -30.70 – 9.22 | | 0.292 |
| **Random Effects** | | | | |  |  |
| σ^2^ | | 17.01 | | |  |  |
| τ_00_ _season_ | | 0.32 | | |  |  |
| τ_00_ _banding_area_ | | 1.78 | | |  |  |
| ICC | | 0.11 | | |  |  |
| N _banding_area_ | | 33 | | |  |  |
| N _season_ | | 43 | | |  |  |
| Observations | | 61841 | | |  |  |
| Marginal R^2^ / Conditional R^2^ | | 0.449 / 0.510 | | |  |  |


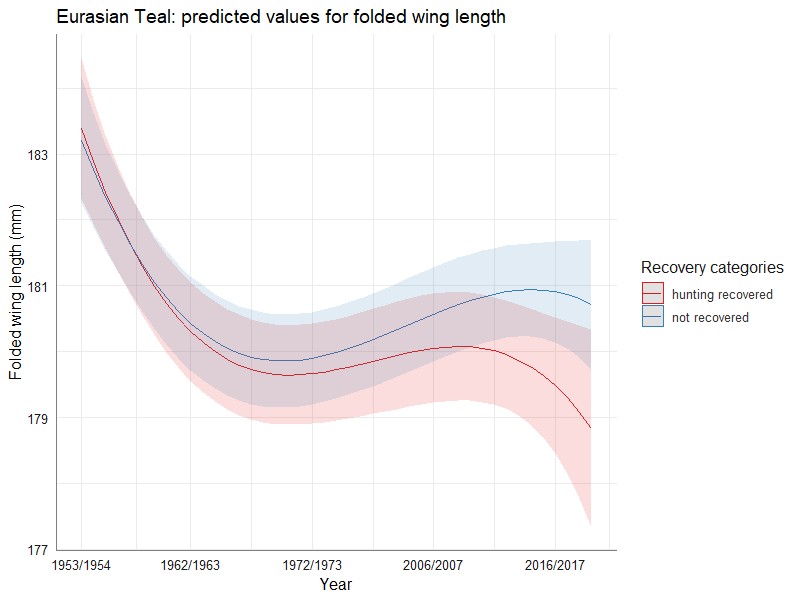


Figure B1: Predicted values of folded wing length as a function of Year and Recovery category in Eurasian Teal (*Anas crecca*).

- **Body mass**

| **Body_mass** | | | | | |  |
| --- | --- | --- | --- | --- | --- | --- |
| ***Predictors*** | ***Estimates*** | | ***std. Error*** | ***CI*** | ***p*** |  |
| **(Intercept)** | 291.03 | | 3.88 | 283.44 – 298.63 | **<0.001** |  |
| **Sex (female)** | -16.11 | | 0.16 | -16.42 – -15.81 | **<0.001** |  |
| **Age(adult)** | 3.79 | | 0.17 | 3.46 – 4.11 | **<0.001** |  |
| **Julian day ^1** | -365.04 | | 49.18 | -461.43 – -268.64 | **<0.001** |  |
| **Julian day ^2** | -1899.61 | | 50.99 | -1999.56 – -1799.67 | **<0.001** |  |
| **Julian day ^3** | 227.92 | | 46.23 | 137.30 – 318.53 | **<0.001** |  |
| **Year^1** | 3666.19 | | 373.47 | 2934.20 – 4398.18 | **<0.001** |  |
| **Year^2** | 795.97 | | 354.18 | 101.80 – 1490.14 | **0.025** |  |
| **Year^3** | -784.09 | | 303.82 | -1379.57 – -188.61 | **0.010** |  |
| **Recovery (Hunting recovery)** | -0.13 | | 0.34 | -0.79 – 0.53 | 0.696 |  |
| **Sex * Age** | -2.02 | | 0.16 | -2.34 – -1.70 | **<0.001** |  |
| **Sex * Julian day ^1** | -340.06 | | 37.71 | -413.97 – -266.14 | **<0.001** |  |
| **Sex * Julian day ^2** | -50.20 | | 38.78 | -126.20 – 25.80 | 0.195 |  |
| **Sex * Julian day ^3** | -87.47 | | 36.78 | -159.55 – -15.39 | **0.017** |  |
| **Sex * Year^1** | 29.87 | | 38.45 | -45.49 – 105.23 | 0.437 |  |
| **Sex * Year^2** | 133.02 | | 37.71 | 59.11 – 206.93 | **<0.001** |  |
| **Sex * Year^3** | 104.36 | | 36.97 | 31.90 – 176.82 | **0.005** |  |
| **Age * Julian day ^1** | -459.14 | | 42.24 | -541.92 – -376.36 | **<0.001** |  |
| **Age * Julian day ^2** | -92.60 | | 42.36 | -175.63 – -9.56 | **0.029** |  |
| **Age * Julian day ^3** | 524.91 | | 40.15 | 446.23 – 603.60 | **<0.001** |  |
| **Age * Year^1** | 95.88 | | 41.42 | 14.69 – 177.07 | **0.021** |  |
| **Age * Year^2** | 96.81 | | 39.19 | 20.00 – 173.62 | **0.013** |  |
| **Age * Year^3** | 45.01 | | 38.32 | -30.09 – 120.12 | 0.240 |  |
| **Julian day ^1 * Year^1** | 74984.21 | | 10687.00 | 54038.07 – 95930.35 | **<0.001** |  |
| **Julian day ^2 * Year^1** | 205226.45 | | 11230.63 | 183214.82 – 227238.09 | **<0.001** |  |
| **Julian day ^3 * Year^1** | 21275.56 | | 9762.49 | 2141.43 – 40409.69 | **0.029** |  |
| **Julian day ^1 * Year^2** | 42850.02 | | 13036.07 | 17299.79 – 68400.24 | **0.001** |  |
| **Julian day ^2 * Year^2** | -73860.83 | | 13617.89 | -100551.41 – -47170.25 | **<0.001** |  |
| **Julian day ^3 * Year^2** | 25412.91 | | 11862.82 | 2162.21 – 48663.62 | **0.032** |  |
| **Julian day ^1 * Year^3** | 44963.46 | | 9862.10 | 25634.11 – 64292.81 | **<0.001** |  |
| **Julian day ^2 * Year^3** | 46308.21 | | 10006.43 | 26695.97 – 65920.45 | **<0.001** |  |
| **Julian day ^3 * Year^3** | -35833.45 | | 9237.33 | -53938.29 – -17728.62 | **<0.001** |  |
| **Year^1 * Recovery** | -165.78 | | 89.38 | -340.96 – 9.40 | 0.064 |  |
| **Year^2 * Recovery** | 163.80 | | 86.14 | -5.02 – 332.63 | 0.057 |  |
| **Year^3 * Recovery** | -88.96 | | 87.41 | -260.28 – 82.36 | 0.309 |  |
| **Random Effects** | | | | | | |
| σ^2^ | | 1249.07 | | | | |
| τ_00_ _season_ | | 94.93 | | | | |
| τ_00_ _banding_area_ | | 227.17 | | | | |
| ICC | | 0.21 | | | | |
| N _banding_area_ | | 33 | | | | |
| N _season_ | | 43 | | | | |
| Observations | | 65030 | | | | |
| Marginal R^2^ / Conditional R^2^ | | 0.254 / 0.407 | | | | |


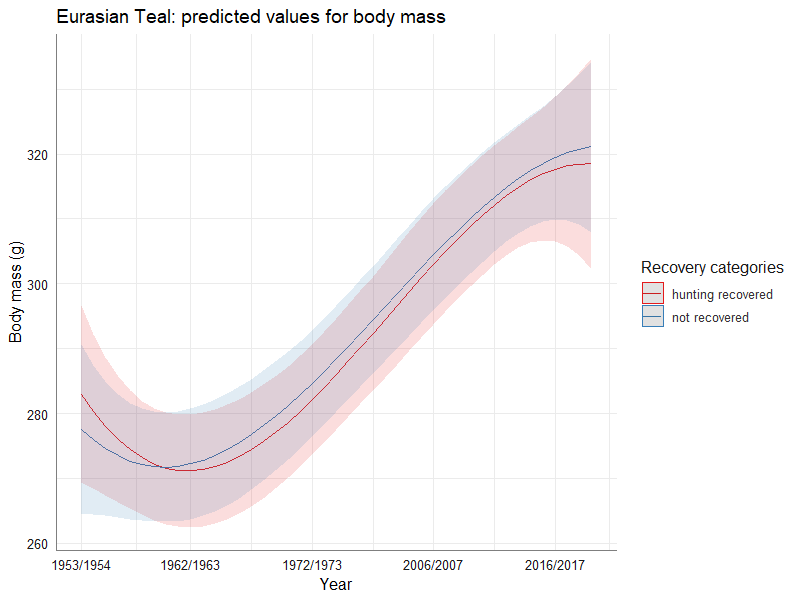


Figure B2: Predicted values of body mass as a function of Year and Recovery category in Eurasian Teal (*Anas crecca*).

- **Body condition index**

| **Body_condition** | | | | |
| --- | --- | --- | --- | --- |
| ***Predictors*** | ***Estimates*** | ***std. Error*** | ***CI*** | ***p*** |
| **(Intercept)** | 305.74 | 4.23 | 297.45 – 314.03 | **<0.001** |
| **Sex (female)** | 10.61 | 0.17 | 10.28 – 10.94 | **<0.001** |
| **Age(adult)** | -3.11 | 0.19 | -3.49 – -2.74 | **<0.001** |
| **Julian day ^1** | -698.35 | 130.04 | -953.22 – -443.49 | **<0.001** |
| **Julian day ^2** | -2418.70 | 123.82 | -2661.38 – -2176.03 | **<0.001** |
| **Julian day ^3** | 137.44 | 113.28 | -84.59 – 359.47 | 0.225 |
| **Year^1** | 4220.80 | 415.45 | 3406.53 – 5035.06 | **<0.001** |
| **Year^2** | -160.50 | 393.18 | -931.12 – 610.13 | 0.683 |
| **Year^3** | -88.65 | 335.77 | -746.74 – 569.44 | 0.792 |
| **Recovery (Hunting recovery)** | 0.02 | 0.45 | -0.86 – 0.89 | 0.969 |
| **Sex * Julian day ^1** | -278.75 | 43.27 | -363.57 – -193.94 | **<0.001** |
| **Sex * Julian day ^2** | -350.86 | 45.62 | -440.28 – -261.45 | **<0.001** |
| **Sex * Julian day ^3** | -59.22 | 42.99 | -143.49 – 25.05 | 0.168 |
| **Sex * Year^1** | 317.55 | 43.48 | 232.33 – 402.76 | **<0.001** |
| **Sex * Year^2** | 78.81 | 44.50 | -8.41 – 166.02 | 0.077 |
| **Sex * Year^3** | 95.29 | 42.26 | 12.47 – 178.11 | **0.024** |
| **Age * Julian day ^1** | -522.40 | 48.86 | -618.17 – -426.63 | **<0.001** |
| **Age * Julian day ^2** | 280.87 | 50.53 | 181.83 – 379.91 | **<0.001** |
| **Age * Julian day ^3** | 474.33 | 46.98 | 382.25 – 566.41 | **<0.001** |
| **Age * Year^1** | 330.18 | 47.50 | 237.08 – 423.28 | **<0.001** |
| **Age * Year^2** | 91.85 | 46.75 | 0.22 – 183.47 | **0.049** |
| **Age * Year^3** | 23.54 | 43.78 | -62.28 – 109.35 | 0.591 |
| **Julian day ^1 * Year^1** | 28585.43 | 12196.05 | 4681.61 – 52489.25 | **0.019** |
| **Julian day ^2 * Year^1** | 203465.60 | 12851.64 | 178276.85 – 228654.36 | **<0.001** |
| **Julian day ^3 * Year^1** | -29506.77 | 11175.39 | -51410.12 – -7603.42 | **0.008** |
| **Julian day ^1 * Year^2** | 152602.22 | 14958.44 | 123284.21 – 181920.23 | **<0.001** |
| **Julian day ^2 * Year^2** | -9713.70 | 15744.97 | -40573.28 – 21145.87 | 0.537 |
| **Julian day ^3 * Year^2** | 80501.39 | 13718.03 | 53614.55 – 107388.23 | **<0.001** |
| **Julian day ^1 * Year^3** | 25045.24 | 11309.48 | 2879.08 – 47211.41 | **0.027** |
| **Julian day ^2 * Year^3** | 55855.17 | 11616.00 | 33088.22 – 78622.11 | **<0.001** |
| **Julian day ^3 * Year^3** | -55064.37 | 10695.98 | -76028.10 – -34100.64 | **<0.001** |
| **Julian day ^1 * Recovery** | -309.98 | 124.31 | -553.61 – -66.34 | **0.013** |
| **Julian day ^2 * Recovery** | -297.71 | 116.38 | -525.81 – -69.61 | **0.011** |
| **Julian day ^3 * Recovery** | -321.85 | 106.82 | -531.21 – -112.49 | **0.003** |
| **Year^1 * Recovery** | 71.45 | 113.95 | -151.89 – 294.79 | 0.531 |
| **Year^2 * Recovery** | 322.44 | 105.01 | 116.63 – 528.25 | **0.002** |
| **Year^3 * Recovery** | 40.01 | 100.80 | -157.57 – 237.58 | 0.691 |
| **Random Effects** | | | | |
| σ^2^ | | 1601.17 | | |
| τ_00_ _season_ | | 114.76 | | |
| τ_00_ _banding_area_ | | 261.96 | | |
| ICC | | 0.19 | | |
| N _banding_area_ | | 33 | | |
| N _season_ | | 43 | | |
| Observations | | 60819 | | |
| Marginal R^2^ / Conditional R^2^ | | 0.190 / 0.344 | | |


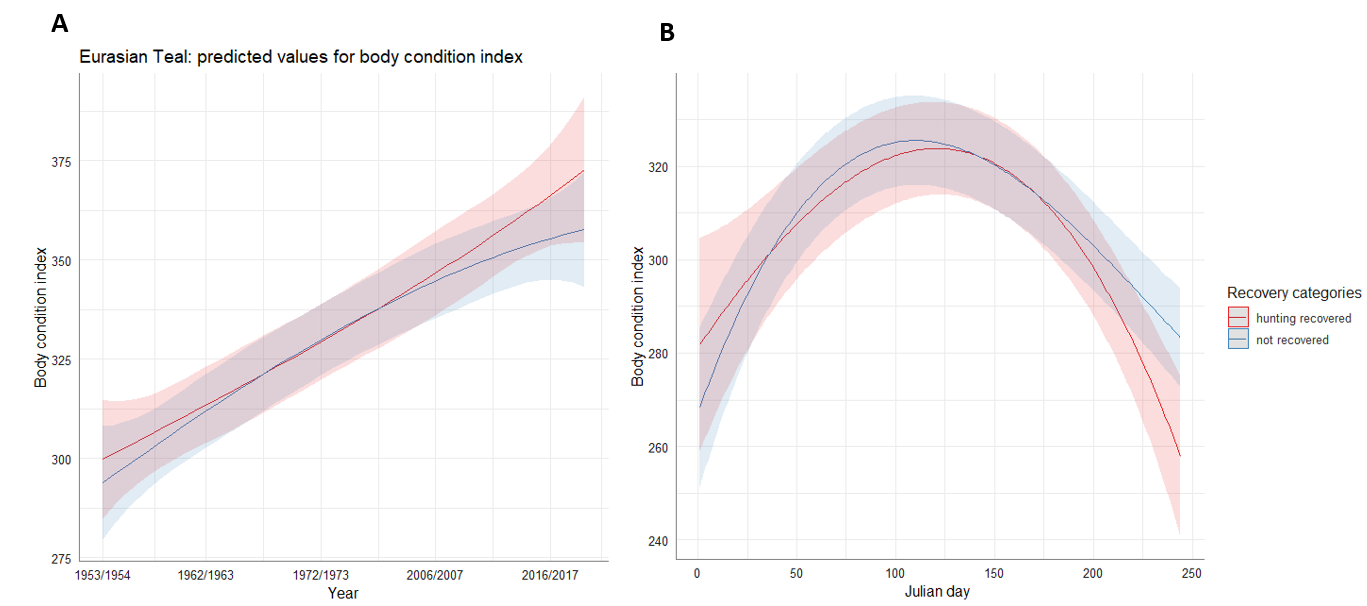


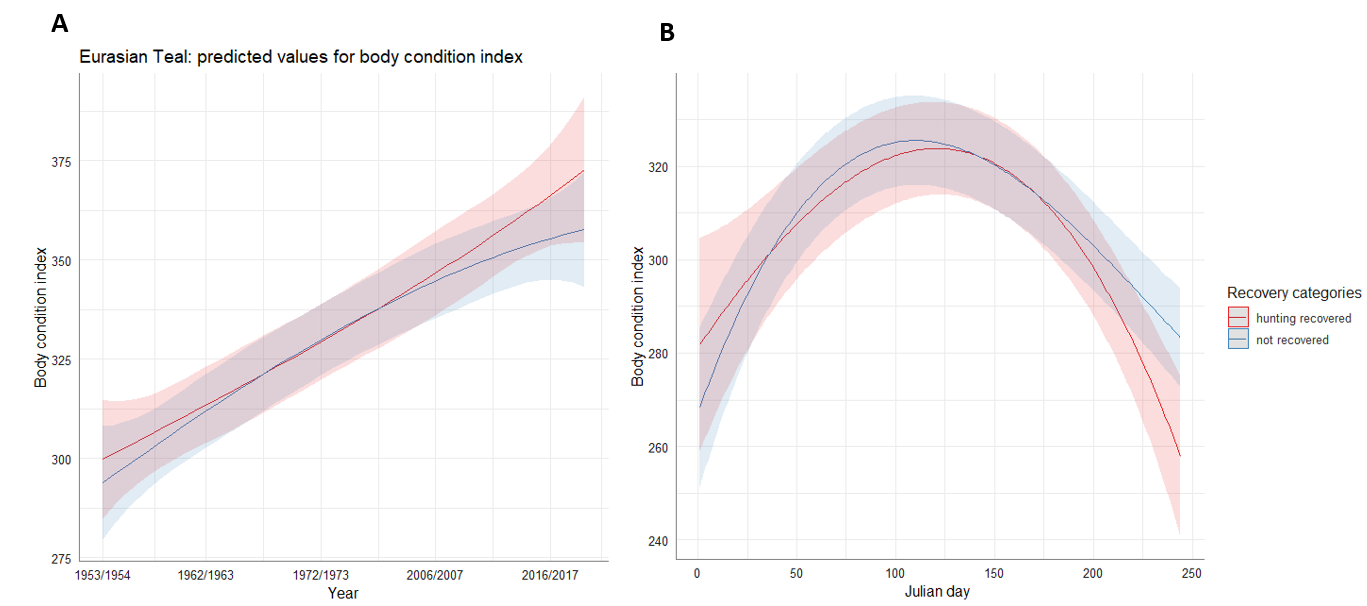


Figure B3: Predicted values of body condition index as a function of Recovery category and Year (A) or Banding day (B) in Eurasian Teal (*Anas crecca*).

- **Mallard**
- **Tarsus**

|  | **tarsus** | | | | |
| --- | --- | --- | --- | --- | --- |
| ***Predictors*** | ***Estimates*** | | ***std. Error*** | ***CI*** | ***p*** |
| **(Intercept)** | 35.04 | | 3.57 | 28.05 – 42.03 | **<0.001** |
| **Sex** | -1.07 | | 0.03 | -1.13 – -1.02 | **<0.001** |
| **Age(adult)** | 0.01 | | 0.03 | -0.05 – 0.07 | 0.786 |
| **Julian day ^1** | -51.16 | | 268.55 | -577.51 – 475.18 | 0.849 |
| **Julian day ^2** | 761.16 | | 277.84 | 216.60 – 1305.71 | **0.006** |
| **Julian day ^3** | 1132.63 | | 306.82 | 531.27 – 1734.00 | **<0.001** |
| **Year^1** | 1284.78 | | 458.35 | 386.43 – 2183.14 | **0.005** |
| **Year^2** | -777.07 | | 247.72 | -1262.60 – -291.54 | **0.002** |
| **Year^3** | 285.39 | | 81.36 | 125.92 – 444.85 | **<0.001** |
| **Age * Julian day ^1** | -8.50 | | 2.84 | -14.06 – -2.94 | **0.003** |
| **Age * Julian day ^2** | 6.31 | | 3.05 | 0.33 – 12.29 | **0.039** |
| **Age * Julian day ^3** | 0.92 | | 3.21 | -5.37 – 7.20 | 0.775 |
| **Julian day ^1 * Year^1** | 6505.95 | | 34340.69 | -60800.56 – 73812.46 | 0.850 |
| **Julian day ^2 * Year^1** | -99189.62 | | 35674.84 | -169111.02 – -29268.23 | **0.005** |
| **Julian day ^3 * Year^1** | -145491.50 | | 39359.99 | -222635.67 – -68347.33 | **<0.001** |
| **Julian day ^1 * Year^2** | -4471.43 | | 18230.59 | -40202.73 – 31259.86 | 0.806 |
| **Julian day ^2 * Year^2** | 56331.53 | | 19177.53 | 18744.26 – 93918.80 | **0.003** |
| **Julian day ^3 * Year^2** | 79045.78 | | 21113.15 | 37664.76 – 120426.79 | **<0.001** |
| **Julian day ^1 * Year^3** | 2044.12 | | 5777.55 | -9279.68 – 13367.92 | 0.723 |
| **Julian day ^2 * Year^3** | -18605.45 | | 6224.01 | -30804.28 – -6406.62 | **0.003** |
| **Julian day ^3 * Year^3** | -24738.93 | | 6803.07 | -38072.71 – -11405.15 | **<0.001** |
| **Random Effects** | | | | | |
| σ^2^ | | 3.88 | | | |
| τ_00_ _banding_area_ | | 0.75 | | | |
| τ_00_ _season_ | | 0.02 | | | |
| ICC | | 0.17 | | | |
| N _banding_area_ | | 20 | | | |
| N _season_ | | 19 | | | |
| Observations | | 5231 | | | |
| Marginal R^2^ / Conditional R^2^ | | 0.203 / 0.335 | | | |

- **Folded wing length**

| **folded_wing** | | | | | |  |
| --- | --- | --- | --- | --- | --- | --- |
| ***Predictors*** | ***Estimates*** | | ***std. Error*** | ***CI*** | ***p*** |  |
| **(Intercept)** | 270.90 | | 0.74 | 269.45 – 272.36 | **<0.001** |  |
| **Sex (female)** | -7.72 | | 0.08 | -7.87 – -7.57 | **<0.001** |  |
| **Age(adult)** | 1.02 | | 0.08 | 0.87 – 1.18 | **<0.001** |  |
| **Julian day ^1** | 49.25 | | 9.63 | 30.38 – 68.12 | **<0.001** |  |
| **Julian day ^2** | -72.95 | | 9.79 | -92.14 – -53.76 | **<0.001** |  |
| **Julian day ^3** | 55.04 | | 8.95 | 37.49 – 72.59 | **<0.001** |  |
| **Year^1** | -64.31 | | 23.13 | -109.64 – -18.99 | **0.005** |  |
| **Year^2** | 71.02 | | 20.05 | 31.72 – 110.32 | **<0.001** |  |
| **Year^3** | 41.61 | | 21.13 | 0.19 – 83.03 | **0.049** |  |
| **Sex * Age** | -0.25 | | 0.08 | -0.40 – -0.11 | **0.001** |  |
| **Sex * Julian day ^1** | -6.34 | | 8.44 | -22.87 – 10.19 | 0.452 |  |
| **Sex * Julian day ^2** | 31.94 | | 8.50 | 15.28 – 48.60 | **<0.001** |  |
| **Sex * Julian day ^3** | -8.89 | | 8.33 | -25.21 – 7.42 | 0.285 |  |
| **Sex * Year^1** | -43.33 | | 8.74 | -60.47 – -26.20 | **<0.001** |  |
| **Sex * Year^2** | 12.61 | | 9.05 | -5.12 – 30.34 | 0.163 |  |
| **Sex * Year^3** | 18.25 | | 8.47 | 1.64 – 34.86 | **0.031** |  |
| **Age * Julian day ^1** | 17.48 | | 8.95 | -0.06 – 35.03 | 0.051 |  |
| **Age * Julian day ^2** | -11.38 | | 9.21 | -29.44 – 6.68 | 0.217 |  |
| **Age * Julian day ^3** | -14.42 | | 8.64 | -31.37 – 2.52 | 0.095 |  |
| **Age * Year^1** | 39.46 | | 9.28 | 21.28 – 57.65 | **<0.001** |  |
| **Age * Year^2** | 18.30 | | 9.23 | 0.21 – 36.38 | **0.047** |  |
| **Age * Year^3** | -46.60 | | 8.62 | -63.50 – -29.71 | **<0.001** |  |
| **Random Effects** | |  |  |  |  |  |
| σ^2^ | | 58.25 | | | | |
| τ_00_ _season_ | | 1.04 | | | | |
| τ_00_ _banding_area_ | | 7.52 | | | | |
| ICC | | 0.13 | | | | |
| N _banding_area_ | | 20 | | | | |
| N _season_ | | 43 | | | | |
| Observations | | 12097 | | | | |
| Marginal R^2^ / Conditional R^2^ | | 0.481 / 0.547 | | | | |

- - **Body mass**

| **body_mass** | | | | | | |
| --- | --- | --- | --- | --- | --- | --- |
| ***Predictors*** | ***Estimates*** | ***std. Error*** | | ***CI*** | | ***p*** |
| **(Intercept)** | 1054.41 | 15.14 | | 1024.72 – 1084.09 | | **<0.001** |
| **Sex (female)** | -62.12 | 1.32 | | -64.71 – -59.54 | | **<0.001** |
| **Age(adult)** | 10.92 | 1.41 | | 8.15 – 13.69 | | **<0.001** |
| **Julian day ^1** | 1129.81 | 196.29 | | 745.10 – 1514.53 | | **<0.001** |
| **Julian day ^2** | -5830.57 | 191.22 | | -6205.35 – -5455.79 | | **<0.001** |
| **Julian day ^3** | -194.76 | 166.56 | | -521.22 – 131.69 | | 0.242 |
| **Year^1** | 5240.25 | 583.46 | | 4096.69 – 6383.80 | | **<0.001** |
| **Year^2** | 268.63 | 519.59 | | -749.76 – 1287.01 | | 0.605 |
| **Year^3** | -2142.25 | 559.61 | | -3239.06 – -1045.44 | | **<0.001** |
| **Sex * Age** | -4.39 | 1.32 | | -6.97 – -1.81 | | **0.001** |
| **Sex * Julian day ^1** | 110.35 | 151.94 | | -187.43 – 408.14 | | 0.468 |
| **Sex * Julian day ^2** | 557.66 | 152.84 | | 258.11 – 857.22 | | **<0.001** |
| **Sex * Julian day ^3** | 102.51 | 146.39 | | -184.41 – 389.42 | | 0.484 |
| **Sex * Year^1** | -393.66 | 159.21 | | -705.71 – -81.61 | | **0.013** |
| **Sex * Year^2** | 202.92 | 161.88 | | -114.35 – 520.20 | | 0.210 |
| **Sex * Year^3** | 177.14 | 152.44 | | -121.63 – 475.92 | | 0.245 |
| **Age * Julian day ^1** | -509.60 | 163.36 | | -829.78 – -189.42 | | **0.002** |
| **Age * Julian day ^2** | 30.07 | 171.91 | | -306.88 – 367.01 | | 0.861 |
| **Age * Julian day ^3** | 314.75 | 155.91 | | 9.18 – 620.33 | | **0.044** |
| **Age * Year^1** | 470.18 | 169.02 | | 138.90 – 801.45 | | **0.005** |
| **Age * Year^2** | 266.05 | 165.25 | | -57.82 – 589.93 | | 0.107 |
| **Age * Year^3** | -521.48 | 154.41 | | -824.13 – -218.84 | | **0.001** |
| **Julian day ^1 * Year^1** | 46939.22 | 18795.88 | | 10099.98 – 83778.47 | | **0.013** |
| **Julian day ^2 * Year^1** | 97305.62 | 18939.31 | | 60185.24 – 134425.99 | | **<0.001** |
| **Julian day ^3 * Year^1** | 49891.75 | 17428.45 | | 15732.61 – 84050.88 | | **0.004** |
| **Julian day ^1 * Year^2** | 75679.42 | 19686.74 | | 37094.13 – 114264.72 | | **<0.001** |
| **Julian day ^2 * Year^2** | 77223.28 | 19635.23 | | 38738.94 – 115707.62 | | **<0.001** |
| **Julian day ^3 * Year^2** | -139184.79 | 18500.55 | | -175445.19 – -102924.38 | | **<0.001** |
| **Julian day ^1 * Year^3** | -38036.51 | 19345.21 | | -75952.43 – -120.60 | | **0.049** |
| **Julian day ^2 * Year^3** | 17378.77 | 19625.96 | | -21087.41 – 55844.95 | | 0.376 |
| **Julian day ^3 * Year^3** | 88546.80 | 18645.03 | | 52003.22 – 125090.39 | | **<0.001** |
| **Random Effects** | | | | |  |  |
| σ^2^ | | | 18617.18 | |  |  |
| τ_00_ _season_ | | | 845.36 | |  |  |
| τ_00_ _banding_area_ | | | 3053.06 | |  |  |
| ICC | | | 0.17 | |  |  |
| N _banding_area_ | | | 20 | |  |  |
| N _season_ | | | 42 | |  |  |
| Observations | | | 12594 | |  |  |
| Marginal R^2^ / Conditional R^2^ | | | 0.282 / 0.406 | |  |  |

- - **Body condition index**

| **body_condition** | | | | | | |
| --- | --- | --- | --- | --- | --- | --- |
| ***Predictors*** | ***Estimates*** | ***std. Error*** | | ***CI*** | | ***p*** |
| **(Intercept)** | 1113.20 | 16.94 | | 1080.00 – 1146.40 | | **<0.001** |
| **Sex (female)** | 58.08 | 1.45 | | 55.24 – 60.92 | | **<0.001** |
| **Age(adult)** | -4.10 | 1.55 | | -7.14 – -1.06 | | **0.008** |
| **Julian day ^1** | 440.32 | 211.64 | | 25.51 – 855.13 | | **0.037** |
| **Julian day ^2** | -4656.74 | 208.26 | | -5064.92 – -4248.57 | | **<0.001** |
| **Julian day ^3** | -1234.41 | 183.07 | | -1593.21 – -875.60 | | **<0.001** |
| **Year^1** | 6037.73 | 523.88 | | 5010.94 – 7064.53 | | **<0.001** |
| **Year^2** | -1093.25 | 463.06 | | -2000.84 – -185.66 | | **0.018** |
| **Year^3** | -2554.18 | 493.55 | | -3521.51 – -1586.84 | | **<0.001** |
| **Sex * Julian day ^1** | 425.40 | 166.14 | | 99.76 – 751.03 | | **0.010** |
| **Sex * Julian day ^2** | -434.46 | 168.12 | | -763.97 – -104.96 | | **0.010** |
| **Sex * Julian day ^3** | 38.21 | 162.96 | | -281.20 – 357.61 | | 0.815 |
| **Sex * Year^1** | 614.82 | 169.49 | | 282.63 – 947.00 | | **<0.001** |
| **Sex * Year^2** | -117.65 | 174.65 | | -459.96 – 224.65 | | 0.501 |
| **Sex * Year^3** | -215.29 | 165.24 | | -539.15 – 108.57 | | 0.193 |
| **Age * Julian day ^1** | -780.44 | 173.86 | | -1121.19 – -439.68 | | **<0.001** |
| **Age * Julian day ^2** | 255.68 | 176.45 | | -90.15 – 601.50 | | 0.147 |
| **Age * Julian day ^3** | 499.68 | 172.67 | | 161.26 – 838.11 | | **0.004** |
| **Julian day ^1 * Year^1** | 44350.54 | 20318.64 | | 4526.73 – 84174.35 | | **0.029** |
| **Julian day ^2 * Year^1** | 99406.08 | 20678.46 | | 58877.05 – 139935.11 | | **<0.001** |
| **Julian day ^3 * Year^1** | 39619.03 | 19070.27 | | 2242.00 – 76996.07 | | **0.038** |
| **Julian day ^1 * Year^2** | 72074.19 | 21026.82 | | 30862.38 – 113285.99 | | **0.001** |
| **Julian day ^2 * Year^2** | 53873.11 | 21325.42 | | 12076.06 – 95670.16 | | **0.012** |
| **Julian day ^3 * Year^2** | -101230.31 | 20223.62 | | -140867.88 – -61592.75 | | **<0.001** |
| **Julian day ^1 * Year^3** | -43761.40 | 20935.73 | | -84794.69 – -2728.12 | | **0.037** |
| **Julian day ^2 * Year^3** | 27158.61 | 21469.40 | | -14920.65 – 69237.87 | | 0.206 |
| **Julian day ^3 * Year^3** | 107208.65 | 20505.46 | | 67018.69 – 147398.61 | | **<0.001** |
| **Random Effects** | | | | |  |  |
| σ^2^ | | | 21980.12 | |  |  |
| τ_00_ _season_ | | | 597.54 | |  |  |
| τ_00_ _banding_area_ | | | 4060.93 | |  |  |
| ICC | | | 0.17 | |  |  |
| N _banding_area_ | | | 20 | |  |  |
| N _season_ | | | 42 | |  |  |
| Observations | | | 11836 | |  |  |
| Marginal R^2^ / Conditional R^2^ | | | 0.210 / 0.348 | |  |  |

- **Common pochard**
- **Tarsus**

| **tarsus** | | | | | | |
| --- | --- | --- | --- | --- | --- | --- |
| ***Predictors*** | ***Estimates*** | | ***std. Error*** | ***CI*** | | ***p*** |
| **(Intercept)** | 37.99 | | 1.38 | 35.29 – 40.69 | | **<0.001** |
| **Sex (female)** | -0.45 | | 0.02 | -0.49 – -0.41 | | **<0.001** |
| **Age(adult)** | -1.01 | | 0.45 | -1.89 – -0.13 | | **0.025** |
| **Julian day ^1** | -24.51 | | 43.29 | -109.36 – 60.35 | | 0.571 |
| **Julian day ^2** | -83.62 | | 37.81 | -157.72 – -9.51 | | **0.027** |
| **Julian day ^3** | -21.36 | | 35.30 | -90.55 – 47.84 | | 0.545 |
| **Year^1** | 162.70 | | 183.16 | -196.30 – 521.69 | | 0.374 |
| **Year^2** | -151.31 | | 130.39 | -406.88 – 104.26 | | 0.246 |
| **Year^3** | 83.13 | | 47.93 | -10.81 – 177.06 | | 0.083 |
| **Recovery (Hunting recovery)** | -0.69 | | 0.88 | -2.40 – 1.03 | | 0.434 |
| **Age * Julian day ^1** | 2.43 | | 2.07 | -1.62 – 6.49 | | 0.240 |
| **Age * Julian day ^2** | -2.69 | | 2.67 | -7.92 – 2.55 | | 0.314 |
| **Age * Julian day ^3** | 6.50 | | 2.22 | 2.14 – 10.86 | | **0.003** |
| **Age * Year^1** | 141.99 | | 62.35 | 19.79 – 264.19 | | **0.023** |
| **Age * Year^2** | -104.98 | | 44.61 | -192.41 – -17.55 | | **0.019** |
| **Age * Year^3** | 37.47 | | 16.86 | 4.41 – 70.52 | | **0.026** |
| **Julian day ^1 * Year^1** | 5458.33 | | 6091.88 | -6481.54 – 17398.20 | | 0.370 |
| **Julian day ^2 * Year^1** | 10198.01 | | 5293.66 | -177.37 – 20573.38 | | 0.054 |
| **Julian day ^3 * Year^1** | 4360.57 | | 4946.23 | -5333.85 – 14055.00 | | 0.378 |
| **Julian day ^1 * Year^2** | -4236.31 | | 4326.82 | -12716.73 – 4244.11 | | 0.328 |
| **Julian day ^2 * Year^2** | -7508.33 | | 3715.60 | -14790.77 – -225.88 | | **0.043** |
| **Julian day ^3 * Year^2** | -3791.42 | | 3504.41 | -10659.93 – 3077.09 | | 0.279 |
| **Julian day ^1 * Year^3** | 2988.16 | | 1755.17 | -451.91 – 6428.22 | | 0.089 |
| **Julian day ^2 * Year^3** | 3510.48 | | 1477.11 | 615.39 – 6405.56 | | **0.017** |
| **Julian day ^3 * Year^3** | 2818.16 | | 1373.12 | 126.90 – 5509.41 | | **0.040** |
| **Year^1 * Recovery** | 100.66 | | 120.83 | -136.17 – 337.49 | | 0.405 |
| **Year^2 * Recovery** | -85.11 | | 85.40 | -252.49 – 82.27 | | 0.319 |
| **Year^3 * Recovery** | 39.25 | | 31.58 | -22.65 – 101.14 | | 0.214 |
| **Random Effects** | | | | |  |  |
| σ^2^ | | 1.26 | | |  |  |
| τ_00_ _season_ | | 0.05 | | |  |  |
| τ_00_ _banding_area_ | | 1.54 | | |  |  |
| ICC | | 0.56 | | |  |  |
| N _banding_area_ | | 12 | | |  |  |
| N _season_ | | 17 | | |  |  |
| Observations | | 3369 | | |  |  |
| Marginal R^2^ / Conditional R^2^ | | 0.107 / 0.606 | | |  |  |


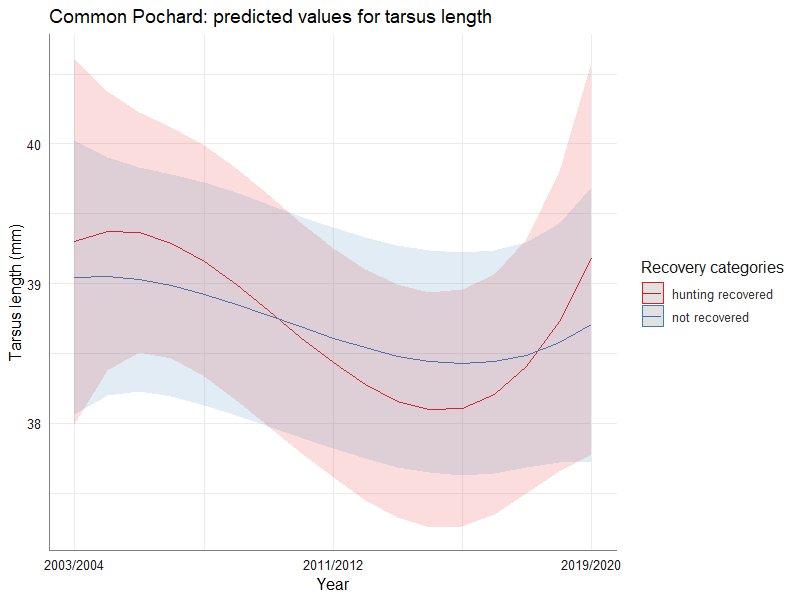


Figure B4: Predicted values of tarsus length as a function of Year and Recovery category in Common Pochard (*Aythya ferina*).

- **Folded wing length**

|  | **folded_wing** | | | | | |
| --- | --- | --- | --- | --- | --- | --- |
| ***Predictors*** | ***Estimates*** | ***std. Error*** | | ***CI*** | ***p*** | |
| **(Intercept)** | 212.82 | 0.76 | | 211.34 – 214.31 | **<0.001** | |
| **Sex (female)** | -3.10 | 0.08 | | -3.25 – -2.95 | **<0.001** | |
| **Age(adult)** | 1.53 | 0.08 | | 1.37 – 1.68 | **<0.001** | |
| **Julian day ^1** | 43.25 | 7.26 | | 29.02 – 57.48 | **<0.001** | |
| **Julian day ^2** | -57.14 | 8.15 | | -73.12 – -41.17 | **<0.001** | |
| **Julian day ^3** | 23.57 | 7.00 | | 9.86 – 37.29 | **0.001** | |
| **Year^1** | 5.32 | 19.90 | | -33.69 – 44.32 | 0.789 | |
| **Year^2** | 32.84 | 8.26 | | 16.65 – 49.02 | **<0.001** | |
| **Year^3** | 9.49 | 9.63 | | -9.39 – 28.37 | 0.324 | |
| **Sex * Age** | -0.34 | 0.08 | | -0.49 – -0.19 | **<0.001** | |
| **Random Effects** | | | | | |  |
| σ^2^ | | | 21.85 | | |  |
| τ_00_ _season_ | | | 0.42 | | |  |
| τ_00_ _banding_area_ | | | 5.26 | | |  |
| ICC | | | 0.21 | | |  |
| N _banding_area_ | | | 13 | | |  |
| N _season_ | | | 39 | | |  |
| Observations | | | 4376 | | |  |
| Marginal R^2^ / Conditional R^2^ | | | 0.329 / 0.468 | | |  |

- **Body mass**

| **body_mass** | | | | | | |
| --- | --- | --- | --- | --- | --- | --- |
| ***Predictors*** | ***Estimates*** | | ***std. Error*** | ***CI*** | ***p*** | |
| **(Intercept)** | 881.92 | | 32.46 | 818.30 – 945.54 | **<0.001** | |
| **Sex (female)** | -17.13 | | 1.44 | -19.95 – -14.32 | **<0.001** | |
| **Age(adult)** | 10.20 | | 1.48 | 7.30 – 13.10 | **<0.001** | |
| **Julian day ^1** | 1762.37 | | 184.34 | 1401.08 – 2123.66 | **<0.001** | |
| **Julian day ^2** | -1936.41 | | 213.46 | -2354.78 – -1518.04 | **<0.001** | |
| **Julian day ^3** | 231.76 | | 160.90 | -83.60 – 547.13 | 0.150 | |
| **Year^1** | 2761.24 | | 732.04 | 1326.47 – 4196.02 | **<0.001** | |
| **Year^2** | -779.38 | | 309.96 | -1386.90 – -171.86 | **0.012** | |
| **Year^3** | -459.68 | | 311.66 | -1070.52 – 151.16 | 0.140 | |
| **Recovery (Hunting recovery)** | -8.64 | | 2.75 | -14.03 – -3.24 | **0.002** | |
| **Sex * Age** | -2.83 | | 1.42 | -5.62 – -0.05 | **0.046** | |
| **Age * Year^1** | 118.14 | | 101.01 | -79.82 – 316.11 | 0.242 | |
| **Age * Year^2** | 240.20 | | 95.97 | 52.11 – 428.30 | **0.012** | |
| **Age * Year^3** | -392.50 | | 95.94 | -580.54 – -204.45 | **<0.001** | |
| **Julian day ^1 * Year^1** | -8838.49 | | 15672.47 | -39555.96 – 21878.98 | 0.573 | |
| **Julian day ^2 * Year^1** | -13821.41 | | 18359.32 | -49805.01 – 22162.19 | 0.452 | |
| **Julian day ^3 * Year^1** | 1163.10 | | 12799.79 | -23924.03 – 26250.23 | 0.928 | |
| **Julian day ^1 * Year^2** | 12541.06 | | 13190.73 | -13312.29 – 38394.41 | 0.342 | |
| **Julian day ^2 * Year^2** | 19156.70 | | 15265.80 | -10763.71 – 49077.12 | 0.210 | |
| **Julian day ^3 * Year^2** | -14705.43 | | 11251.58 | -36758.12 – 7347.27 | 0.191 | |
| **Julian day ^1 * Year^3** | -15242.36 | | 13557.95 | -41815.45 – 11330.72 | 0.261 | |
| **Julian day ^2 * Year^3** | -26167.74 | | 14589.60 | -54762.83 – 2427.35 | 0.073 | |
| **Julian day ^3 * Year^3** | 37378.55 | | 11933.64 | 13989.06 – 60768.05 | **0.002** | |
| **Random Effects** | | | | | |  |
| σ^2^ | | 7328.96 | | | |  |
| τ_00_ _season_ | | 830.37 | | | |  |
| τ_00_ _banding_area_ | | 11712.41 | | | |  |
| ICC | | 0.63 | | | |  |
| N _banding_area_ | | 13 | | | |  |
| N _season_ | | 39 | | | |  |
| Observations | | 4357 | | | |  |
| Marginal R^2^ / Conditional R^2^ | | 0.132 / 0.680 | | | |  |

- - **Body condition index**

| **body_condition** | | | | | | |
| --- | --- | --- | --- | --- | --- | --- |
| ***Predictors*** | ***Estimates*** | | ***std. Error*** | ***CI*** | | ***p*** |
| **(Intercept)** | 944.42 | | 49.23 | 847.93 – 1040.91 | | **<0.001** |
| **Sex (female)** | 42.05 | | 1.73 | 38.67 – 45.44 | | **<0.001** |
| **Age(adult)** | -22.83 | | 2.10 | -26.95 – -18.71 | | **<0.001** |
| **Julian day ^1** | 1105.56 | | 257.21 | 601.44 – 1609.68 | | **<0.001** |
| **Julian day ^2** | -1085.09 | | 324.23 | -1720.56 – -449.62 | | **0.001** |
| **Julian day ^3** | 7.83 | | 238.99 | -460.57 – 476.24 | | 0.974 |
| **Year^1** | 2428.47 | | 799.53 | 861.41 – 3995.53 | | **0.002** |
| **Year^2** | -1509.98 | | 343.04 | -2182.33 – -837.63 | | **<0.001** |
| **Year^3** | -526.20 | | 348.60 | -1209.45 – 157.04 | | 0.131 |
| **Age * Julian day ^1** | 376.98 | | 196.13 | -7.43 – 761.38 | | 0.055 |
| **Age * Julian day ^2** | -760.77 | | 263.38 | -1276.99 – -244.56 | | **0.004** |
| **Age * Julian day ^3** | 636.13 | | 209.89 | 224.75 – 1047.50 | | **0.002** |
| **Age * Year^1** | -105.63 | | 140.40 | -380.81 – 169.56 | | 0.452 |
| **Age * Year^2** | 142.17 | | 121.89 | -96.74 – 381.07 | | 0.243 |
| **Age * Year^3** | -341.76 | | 123.86 | -584.53 – -98.98 | | **0.006** |
| **Julian day ^1 * Year^1** | 15115.27 | | 19877.74 | -23844.39 – 54074.93 | | 0.447 |
| **Julian day ^2 * Year^1** | -43497.21 | | 23357.16 | -89276.41 – 2281.99 | | 0.063 |
| **Julian day ^3 * Year^1** | 19461.56 | | 16308.78 | -12503.06 – 51426.19 | | 0.233 |
| **Julian day ^1 * Year^2** | 30980.31 | | 16819.91 | -1986.10 – 63946.72 | | 0.065 |
| **Julian day ^2 * Year^2** | -3362.34 | | 19383.83 | -41353.96 – 34629.27 | | 0.862 |
| **Julian day ^3 * Year^2** | 3434.82 | | 14315.58 | -24623.20 – 31492.83 | | 0.810 |
| **Julian day ^1 * Year^3** | -16821.67 | | 17170.13 | -50474.51 – 16831.17 | | 0.327 |
| **Julian day ^2 * Year^3** | -10813.56 | | 18526.06 | -47123.97 – 25496.84 | | 0.559 |
| **Julian day ^3 * Year^3** | 34486.76 | | 15148.13 | 4796.97 – 64176.56 | | **0.023** |
| **Random Effects** | | | | |  |  |
| σ^2^ | | 11851.99 | | |  |  |
| τ_00_ _season_ | | 860.68 | | |  |  |
| τ_00_ _banding_area_ | | 28451.67 | | |  |  |
| ICC | | 0.71 | | |  |  |
| N _banding_area_ | | 13 | | |  |  |
| N _season_ | | 39 | | |  |  |
| Observations | | 4339 | | |  |  |
| Marginal R^2^ / Conditional R^2^ | | 0.079 / 0.735 | | |  |  |

- **Tufted Duck**
  - **Tarsus**

| **tarsus** | | | | | | |
| --- | --- | --- | --- | --- | --- | --- |
| ***Predictors*** | ***Estimates*** | | ***std. Error*** | ***CI*** | ***p*** | |
| **(Intercept)** | 48.29 | | 4.53 | 39.41 – 57.17 | **<0.001** | |
| **Sex (female)** | -0.31 | | 0.03 | -0.37 – -0.25 | **<0.001** | |
| **Age(adult)** | 0.10 | | 0.03 | 0.03 – 0.16 | **0.002** | |
| **Julian day ^1** | -25.50 | | 222.09 | -460.78 – 409.78 | 0.909 | |
| **Julian day ^2** | 168.91 | | 197.66 | -218.49 – 556.32 | 0.393 | |
| **Julian day ^3** | 18.96 | | 203.95 | -380.77 – 418.69 | 0.926 | |
| **Year^1** | -928.64 | | 309.65 | -1535.55 – -321.74 | **0.003** | |
| **Year^2** | 567.96 | | 190.76 | 194.08 – 941.83 | **0.003** | |
| **Year^3** | -152.71 | | 57.67 | -265.73 – -39.68 | **0.008** | |
| **Julian day ^1 * Year^1** | 2587.48 | | 15401.59 | -27599.08 – 32774.04 | 0.867 | |
| **Julian day ^2 * Year^1** | -10618.54 | | 13549.45 | -37174.96 – 15937.89 | 0.433 | |
| **Julian day ^3 * Year^1** | -1481.82 | | 13819.18 | -28566.92 – 25603.29 | 0.915 | |
| **Julian day ^1 * Year^2** | -1573.57 | | 9627.77 | -20443.65 – 17296.50 | 0.870 | |
| **Julian day ^2 * Year^2** | 5790.79 | | 8356.12 | -10586.89 – 22168.48 | 0.488 | |
| **Julian day ^3 * Year^2** | 1150.76 | | 8371.01 | -15256.12 – 17557.64 | 0.891 | |
| **Julian day ^1 * Year^3** | 1263.98 | | 3122.96 | -4856.91 – 7384.87 | 0.686 | |
| **Julian day ^2 * Year^3** | -738.42 | | 2579.14 | -5793.45 – 4316.61 | 0.775 | |
| **Julian day ^3 * Year^3** | -257.65 | | 2463.82 | -5086.64 – 4571.34 | 0.917 | |
| **Random Effects** | | | | | |  |
| σ^2^ | | 1.28 | | | |  |
| τ_00_ _season_ | | 0.03 | | | |  |
| ICC | | 0.02 | | | |  |
| N _season_ | | 16 | | | |  |
| Observations | | 1659 | | | |  |
| Marginal R^2^ / Conditional R^2^ | | 0.120 / 0.138 | | | |  |

- **Folded wing length**

| **folded_wing** | | | | | |
| --- | --- | --- | --- | --- | --- |
| ***Predictors*** | ***Estimates*** | | ***std. Error*** | ***CI*** | ***p*** |
| **(Intercept)** | 204.10 | | 0.48 | 203.17 – 205.04 | **<0.001** |
| **Sex (female)** | -3.31 | | 0.08 | -3.47 – -3.15 | **<0.001** |
| **Age** | 0.94 | | 0.08 | 0.79 – 1.10 | **<0.001** |
| **Julian day ^1** | 6.75 | | 8.32 | -9.55 – 23.04 | 0.417 |
| **Julian day ^2** | -4.42 | | 9.94 | -23.90 – 15.06 | 0.657 |
| **Julian day ^3** | 4.44 | | 8.25 | -11.73 – 20.61 | 0.590 |
| **Year^1** | -54.69 | | 20.11 | -94.09 – -15.28 | **0.007** |
| **Year^2** | 52.49 | | 8.61 | 35.62 – 69.37 | **<0.001** |
| **Year^3** | -23.06 | | 9.50 | -41.67 – -4.44 | **0.015** |
| **Sex * Age** | -0.34 | | 0.08 | -0.49 – -0.19 | **<0.001** |
| **Julian day ^1 * Year^1** | -672.48 | | 526.64 | -1704.67 – 359.72 | 0.202 |
| **Julian day ^2 * Year^1** | -603.43 | | 468.06 | -1520.81 – 313.95 | 0.197 |
| **Julian day ^3 * Year^1** | 281.38 | | 365.53 | -435.04 – 997.80 | 0.441 |
| **Julian day ^1 * Year^2** | -1593.27 | | 564.51 | -2699.69 – -486.84 | **0.005** |
| **Julian day ^2 * Year^2** | -1203.20 | | 551.89 | -2284.89 – -121.51 | **0.029** |
| **Julian day ^3 * Year^2** | 527.50 | | 445.70 | -346.06 – 1401.06 | 0.237 |
| **Julian day ^1 * Year^3** | -784.69 | | 520.88 | -1805.59 – 236.21 | 0.132 |
| **Julian day ^2 * Year^3** | -1096.08 | | 561.28 | -2196.18 – 4.01 | 0.051 |
| **Julian day ^3 * Year^3** | -198.23 | | 456.47 | -1092.89 – 696.43 | 0.664 |
| **Random Effects** | | | | | |
| σ^2^ | | 19.40 | | | |
| τ_00_ _season_ | | 0.16 | | | |
| τ_00_ _banding_area_ | | 0.94 | | | |
| ICC | | 0.05 | | | |
| N _banding_area_ | | 12 | | | |
| N _season_ | | 37 | | | |
| Observations | | 4644 | | | |
| Marginal R^2^ / Conditional R^2^ | | 0.345 / 0.380 | | | |

- **Body mass**

|  | **body_mass** | | | | | |
| --- | --- | --- | --- | --- | --- | --- |
| ***Predictors*** | | ***Estimates*** | ***std. Error*** | | ***CI*** | ***p*** |
| **(Intercept)** | | 756.10 | 15.33 | | 726.05 – 786.16 | **<0.001** |
| **Sex (female)** | | -24.22 | 1.14 | | -26.46 – -21.99 | **<0.001** |
| **Age(adult)** | | 7.78 | 1.33 | | 5.18 – 10.38 | **<0.001** |
| **Julian day ^1** | | 685.21 | 147.09 | | 396.92 – 973.49 | **<0.001** |
| **Julian day ^2** | | -977.04 | 171.93 | | -1314.03 – -640.06 | **<0.001** |
| **Julian day ^3** | | -240.22 | 146.96 | | -528.26 – 47.82 | 0.102 |
| **Year^1** | | 1700.18 | 594.19 | | 535.58 – 2864.78 | **0.004** |
| **Year^2** | | -213.27 | 242.52 | | -688.61 – 262.06 | 0.379 |
| **Year^3** | | -695.04 | 281.66 | | -1247.08 – -142.99 | **0.014** |
| **Age * Julian day ^1** | | -297.23 | 93.35 | | -480.18 – -114.27 | **0.001** |
| **Age * Julian day ^2** | | 110.77 | 93.65 | | -72.78 – 294.32 | 0.237 |
| **Age * Julian day ^3** | | -213.53 | 89.75 | | -389.44 – -37.62 | **0.017** |
| **Julian day ^1 * Year^1** | | -50414.42 | 9365.37 | | -68770.20 – -32058.63 | **<0.001** |
| **Julian day ^2 * Year^1** | | -12690.87 | 8554.25 | | -29456.88 – 4075.15 | 0.138 |
| **Julian day ^3 * Year^1** | | -6525.73 | 5933.02 | | -18154.24 – 5102.77 | 0.271 |
| **Julian day ^1 * Year^2** | | -22419.03 | 9778.27 | | -41584.08 – -3253.99 | **0.022** |
| **Julian day ^2 * Year^2** | | -24460.22 | 9758.56 | | -43586.65 – -5333.79 | **0.012** |
| **Julian day ^3 * Year^2** | | -1455.76 | 7332.03 | | -15826.26 – 12914.75 | 0.843 |
| **Julian day ^1 * Year^3** | | 34417.36 | 9202.55 | | 16380.70 – 52454.02 | **<0.001** |
| **Julian day ^2 * Year^3** | | 14392.70 | 9472.68 | | -4173.40 – 32958.81 | 0.129 |
| **Julian day ^3 * Year^3** | | -12972.51 | 7844.57 | | -28347.58 – 2402.56 | 0.098 |
| **Random Effects** | | | | | | |
| σ^2^ | | | | 5259.55 | | |
| τ_00_ _season_ | | | | 283.61 | | |
| τ_00_ _banding_area_ | | | | 1159.34 | | |
| ICC | | | | 0.22 | | |
| N _banding_area_ | | | | 13 | | |
| N _season_ | | | | 37 | | |
| Observations | | | | 4628 | | |
| Marginal R^2^ / Conditional R^2^ | | | | 0.216 / 0.385 | | |

- - **Body condition index**

| **body_condition** | | | | | | |
| --- | --- | --- | --- | --- | --- | --- |
| ***Predictors*** | ***Estimates*** | | ***std. Error*** | | ***CI*** | ***p*** |
| **(Intercept)** | 743.03 | | 18.98 | | 705.83 – 780.23 | **<0.001** |
| **Sex (female)** | 29.18 | | 1.55 | | 26.15 – 32.21 | **<0.001** |
| **Age(adult)** | -7.40 | | 1.53 | | -10.39 – -4.41 | **<0.001** |
| **Julian day ^1** | 719.39 | | 165.21 | | 395.58 – 1043.21 | **<0.001** |
| **Julian day ^2** | -892.57 | | 195.47 | | -1275.68 – -509.45 | **<0.001** |
| **Julian day ^3** | -211.20 | | 163.39 | | -531.44 – 109.04 | 0.196 |
| **Year^1** | 2644.84 | | 656.48 | | 1358.17 – 3931.52 | **<0.001** |
| **Year^2** | -1003.38 | | 256.84 | | -1506.79 – -499.97 | **<0.001** |
| **Year^3** | -286.12 | | 301.69 | | -877.42 – 305.18 | 0.343 |
| **Sex * Age** | 4.38 | | 1.51 | | 1.42 – 7.34 | **0.004** |
| **Julian day ^1 * Year^1** | -36120.30 | | 10613.63 | | -56922.63 – -15317.98 | **0.001** |
| **Julian day ^2 * Year^1** | -8443.87 | | 9474.15 | | -27012.86 – 10125.11 | 0.373 |
| **Julian day ^3 * Year^1** | -5963.19 | | 7128.57 | | -19934.92 – 8008.55 | 0.403 |
| **Julian day ^1 * Year^2** | 3976.94 | | 11217.37 | | -18008.70 – 25962.58 | 0.723 |
| **Julian day ^2 * Year^2** | 818.85 | | 11173.52 | | -21080.84 – 22718.55 | 0.942 |
| **Julian day ^3 * Year^2** | -9261.48 | | 8727.43 | | -26366.93 – 7843.97 | 0.289 |
| **Julian day ^1 * Year^3** | 55273.93 | | 10541.14 | | 34613.68 – 75934.18 | **<0.001** |
| **Julian day ^2 * Year^3** | 31406.23 | | 10967.91 | | 9909.51 – 52902.94 | **0.004** |
| **Julian day ^3 * Year^3** | -3065.71 | | 8973.18 | | -20652.81 – 14521.40 | 0.733 |
| **Random Effects** | | | |  |  |  |
| σ^2^ | | 7069.44 | |  |  |  |
| τ_00_ _season_ | | 286.91 | |  |  |  |
| τ_00_ _banding_area_ | | 2184.12 | |  |  |  |
| ICC | | 0.26 | |  |  |  |
| N _banding_area_ | | 12 | |  |  |  |
| N _season_ | | 37 | |  |  |  |
| Observations | | 4616 | |  |  |  |
| Marginal R^2^ / Conditional R^2^ | | 0.180 / 0.392 | |  |  |  |

- **Eurasian Coot**
  - - **Folded wing length**

| **folded_wing** | | | | | | |
| --- | --- | --- | --- | --- | --- | --- |
| ***Predictors*** | ***Estimates*** | | ***std. Error*** | ***CI*** | ***p*** | |
| **(Intercept)** | 209.69 | | 0.39 | 208.92 – 210.46 | **<0.001** | |
| **Sex (female)** | -9.99 | | 0.13 | -10.25 – -9.74 | **<0.001** | |
| **Age(adult)** | 0.52 | | 0.14 | 0.25 – 0.79 | **<0.001** | |
| **Julian day ^1** | 13.60 | | 6.45 | 0.96 – 26.25 | **0.035** | |
| **Julian day ^2** | -5.07 | | 7.50 | -19.78 – 9.64 | 0.499 | |
| **Julian day ^3** | -9.13 | | 7.47 | -23.78 – 5.52 | 0.222 | |
| **Year^1** | -32.44 | | 13.26 | -58.43 – -6.46 | **0.014** | |
| **Year^2** | 42.35 | | 12.53 | 17.78 – 66.92 | **0.001** | |
| **Year^3** | 14.95 | | 10.16 | -4.97 – 34.87 | 0.141 | |
| **Sex * Age** | 0.28 | | 0.13 | 0.02 – 0.53 | **0.033** | |
| **Sex * Year^1** | -44.58 | | 5.23 | -54.83 – -34.33 | **<0.001** | |
| **Sex * Year^2** | 54.29 | | 5.32 | 43.87 – 64.71 | **<0.001** | |
| **Sex * Year^3** | -8.10 | | 5.51 | -18.90 – 2.69 | 0.141 | |
| **Julian day ^1 * Year^1** | -31.54 | | 302.81 | -625.04 – 561.95 | 0.917 | |
| **Julian day ^2 * Year^1** | 666.73 | | 259.59 | 157.95 – 1175.52 | **0.010** | |
| **Julian day ^3 * Year^1** | -256.26 | | 232.58 | -712.12 – 199.59 | 0.271 | |
| **Julian day ^1 * Year^2** | -298.56 | | 318.07 | -921.96 – 324.83 | 0.348 | |
| **Julian day ^2 * Year^2** | -408.11 | | 289.92 | -976.34 – 160.12 | 0.159 | |
| **Julian day ^3 * Year^2** | 74.01 | | 316.89 | -547.07 – 695.10 | 0.815 | |
| **Julian day ^1 * Year^3** | 54.07 | | 248.38 | -432.75 – 540.89 | 0.828 | |
| **Julian day ^2 * Year^3** | -129.42 | | 229.97 | -580.15 – 321.31 | 0.574 | |
| **Julian day ^3 * Year^3** | -112.91 | | 226.55 | -556.93 – 331.12 | 0.618 | |
| **Random Effects** | | | | | |  |
| σ^2^ | | 23.75 | | | |  |
| τ_00_ _season_ | | 2.37 | | | |  |
| ICC | | 0.09 | | | |  |
| N _season_ | | 30 | | | |  |
| Observations | | 1845 | | | |  |
| Marginal R^2^ / Conditional R^2^ | | 0.797 / 0.816 | | | |  |

- - - **Body mass**

| **body_mass** | | | | | | |
| --- | --- | --- | --- | --- | --- | --- |
| ***Predictors*** | ***Estimates*** | | ***std. Error*** | ***CI*** | | *p* |
| **(Intercept)** | 625.33 | | 10.94 | 603.89 – 646.77 | | **<0.001** |
| **Sex (female)** | -76.56 | | 2.50 | -81.47 – -71.66 | | **<0.001** |
| **Age(adult)** | 22.43 | | 6.00 | 10.67 – 34.18 | | **<0.001** |
| **Julian day ^1** | -428.10 | | 127.97 | -678.92 – -177.28 | | **0.001** |
| **Julian day ^2** | 45.68 | | 144.53 | -237.60 – 328.95 | | 0.752 |
| **Julian day ^3** | -12.03 | | 145.16 | -296.54 – 272.48 | | 0.934 |
| **Year^1** | 360.44 | | 325.93 | -278.37 – 999.25 | | 0.269 |
| **Year^2** | 640.89 | | 283.64 | 84.97 – 1196.81 | | **0.024** |
| **Year^3** | 47.37 | | 235.95 | -415.09 – 509.83 | | 0.841 |
| **Recovery (Hunting recovery)** | -5.30 | | 6.00 | -17.07 – 6.47 | | 0.378 |
| **Sex * Age** | -6.61 | | 2.50 | -11.51 – -1.70 | | **0.008** |
| **Sex * Year^1** | -531.19 | | 107.56 | -742.01 – -320.38 | | **<0.001** |
| **Sex * Year^2** | 135.14 | | 104.91 | -70.48 – 340.75 | | 0.198 |
| **Sex * Year^3** | -101.33 | | 106.97 | -310.99 – 108.32 | | 0.343 |
| **Age * Year^1** | -482.48 | | 132.20 | -741.59 – -223.37 | | **<0.001** |
| **Age * Year^2** | 133.05 | | 114.36 | -91.08 – 357.19 | | 0.245 |
| **Age * Year^3** | 212.33 | | 121.90 | -26.59 – 451.25 | | 0.082 |
| **Age * Recovery** | -13.08 | | 5.94 | -24.73 – -1.43 | | **0.028** |
| **Julian day ^1 * Year^1** | 16543.89 | | 5964.78 | 4853.14 – 28234.64 | | **0.006** |
| **Julian day ^2 * Year^1** | 3863.93 | | 5023.20 | -5981.37 – 13709.22 | | 0.442 |
| **Julian day ^3 * Year^1** | -12236.34 | | 4500.96 | -21058.06 – -3414.61 | | **0.007** |
| **Julian day ^1 * Year^2** | -12729.91 | | 6072.60 | -24631.99 – -827.83 | | **0.036** |
| **Julian day ^2 * Year^2** | -11934.30 | | 5405.07 | -22528.05 – -1340.55 | | **0.027** |
| **Julian day ^3 * Year^2** | 2494.45 | | 5950.64 | -9168.58 – 14157.48 | | 0.675 |
| **Julian day ^1 * Year^3** | -4475.90 | | 4892.20 | -14064.42 – 5112.63 | | 0.360 |
| **Julian day ^2 * Year^3** | 444.57 | | 4549.29 | -8471.88 – 9361.02 | | 0.922 |
| **Julian day ^3 * Year^3** | 2093.86 | | 4555.35 | -6834.46 – 11022.19 | | 0.646 |
| **Random Effects** | |  |  |  |  |  |
| σ^2^ | | 7863.53 | | |  |  |
| τ_00_ _season_ | | 1377.81 | | |  |  |
| ICC | | 0.15 | | |  |  |
| N _season_ | | 29 | | |  |  |
| Observations | | 1690 | | |  |  |
| Marginal R^2^ / Conditional R^2^ | | 0.480 / 0.557 | | |  |  |


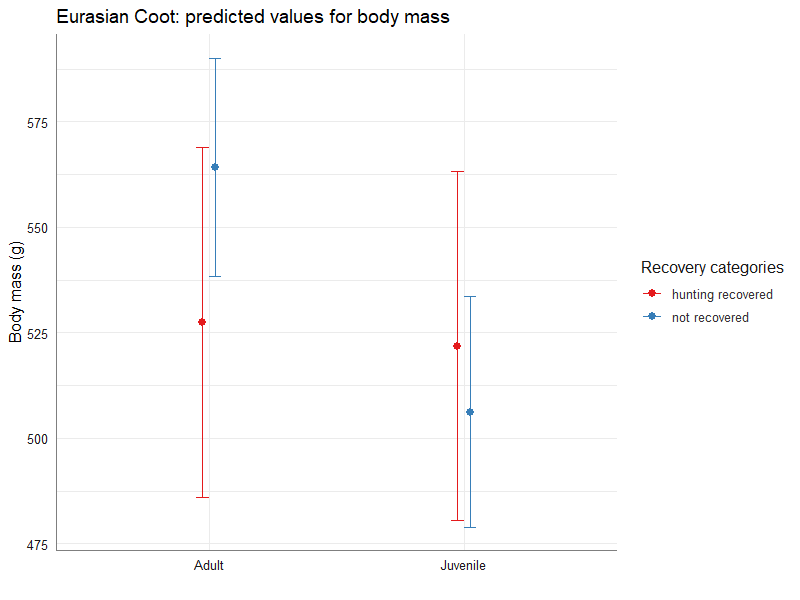


Figure B5: Predicted values of body mass as a function of Ageand Recovery category in Eurasian Coot (*Fulica atra*).

- **Body condition index**

| **body_condition** | | | | | | |
| --- | --- | --- | --- | --- | --- | --- |
| ***Predictors*** | ***Estimates*** | | ***std. Error*** | ***CI*** | | ***p*** |
| **(Intercept)** | 646.60 | | 10.91 | 625.22 – 667.98 | | **<0.001** |
| **Sex (female)** | 39.55 | | 2.49 | 34.66 – 44.43 | | **<0.001** |
| **Age(adult)** | 12.58 | | 6.45 | -0.06 – 25.23 | | 0.051 |
| **Julian day ^1** | -443.15 | | 133.72 | -705.23 – -181.07 | | **0.001** |
| **Julian day ^2** | 226.61 | | 123.77 | -15.98 – 469.20 | | 0.067 |
| **Julian day ^3** | 394.78 | | 117.72 | 164.05 – 625.50 | | **0.001** |
| **Year^1** | 445.91 | | 310.26 | -162.19 – 1054.01 | | 0.151 |
| **Year^2** | 92.65 | | 260.88 | -418.67 – 603.97 | | 0.722 |
| **Year^3** | -195.30 | | 205.72 | -598.51 – 207.92 | | 0.342 |
| **Recovery (Hunting recovery)** | -9.72 | | 6.37 | -22.21 – 2.77 | | 0.127 |
| **Sex * Year^1** | 186.41 | | 109.15 | -27.52 – 400.34 | | 0.088 |
| **Sex * Year^2** | -386.69 | | 105.47 | -593.41 – -179.97 | | **<0.001** |
| **Sex * Year^3** | -13.21 | | 106.31 | -221.58 – 195.16 | | 0.901 |
| **Age * Julian day ^1** | -274.61 | | 121.25 | -512.26 – -36.96 | | **0.024** |
| **Age * Julian day ^2** | -288.48 | | 119.50 | -522.70 – -54.26 | | **0.016** |
| **Age * Julian day ^3** | -158.37 | | 112.65 | -379.15 – 62.41 | | 0.160 |
| **Age * Recovery** | -15.43 | | 6.33 | -27.84 – -3.02 | | **0.015** |
| **Random Effects** | | | | |  |  |
| σ^2^ | | 8961.32 | | |  |  |
| τ_00_ _season_ | | 1306.92 | | |  |  |
| ICC | | 0.13 | | |  |  |
| N _season_ | | 29 | | |  |  |
| Observations | | 1687 | | |  |  |
| Marginal R^2^ / Conditional R^2^ | | 0.168 / 0.274 | | |  |  |


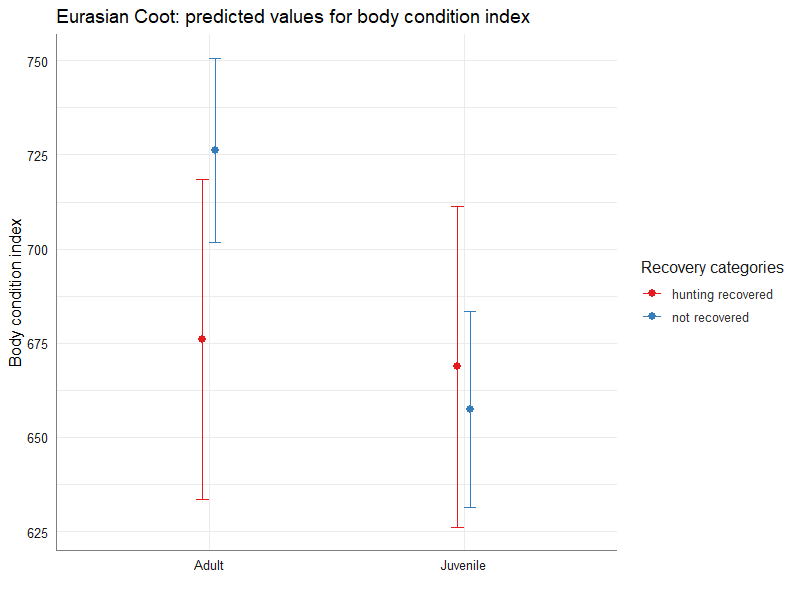


Figure B6: Predicted values of body condition index as a function of Age and Recovery category in Eurasian Coot (*Fulica atra*).

- **Common Snipe**
- **Tarsus**

| **tarsus** | | | | | | |
| --- | --- | --- | --- | --- | --- | --- |
| ***Predictors*** | ***Estimates*** | | ***std. Error*** | | ***CI*** | ***p*** |
| **(Intercept)** | 34.07 | | 0.14 | | 33.80 – 34.34 | **<0.001** |
| **Sex (female)** | 0.16 | | 0.01 | | 0.14 – 0.19 | **<0.001** |
| **Age(adult)** | 0.07 | | 0.01 | | 0.04 – 0.10 | **<0.001** |
| **Julian day ^1** | -9.28 | | 2.49 | | -14.15 – -4.41 | **<0.001** |
| **Julian day ^2** | -9.54 | | 2.26 | | -13.98 – -5.10 | **<0.001** |
| **Julian day ^3** | 2.16 | | 2.07 | | -1.90 – 6.22 | 0.297 |
| **Year^1** | -34.94 | | 10.70 | | -55.92 – -13.96 | **0.001** |
| **Year^2** | -24.90 | | 10.91 | | -46.29 – -3.51 | **0.023** |
| **Year^3** | 15.49 | | 7.75 | | 0.30 – 30.67 | **0.046** |
| **Julian day ^1 * Year^1** | -878.78 | | 479.18 | | -1817.95 – 60.39 | 0.067 |
| **Julian day ^2 * Year^1** | 1878.87 | | 439.10 | | 1018.25 – 2739.50 | **<0.001** |
| **Julian day ^3 * Year^1** | 147.25 | | 400.84 | | -638.38 – 932.88 | 0.713 |
| **Julian day ^1 * Year^2** | 876.17 | | 414.79 | | 63.20 – 1689.14 | **0.035** |
| **Julian day ^2 * Year^2** | -204.95 | | 389.52 | | -968.39 – 558.48 | 0.599 |
| **Julian day ^3 * Year^2** | -448.46 | | 327.25 | | -1089.85 – 192.93 | 0.171 |
| **Julian day ^1 * Year^3** | -400.23 | | 386.20 | | -1157.17 – 356.70 | 0.300 |
| **Julian day ^2 * Year^3** | -48.18 | | 392.51 | | -817.49 – 721.13 | 0.902 |
| **Julian day ^3 * Year^3** | -61.60 | | 317.32 | | -683.54 – 560.34 | 0.846 |
| **Random Effects** | | | |  |  |  |
| σ^2^ | | 2.18 | |  |  |  |
| τ_00_ _banding_area_ | | 0.44 | |  |  |  |
| τ_00_ _season_ | | 0.18 | |  |  |  |
| ICC | | 0.22 | |  |  |  |
| N _banding_area_ | | 61 | |  |  |  |
| N _season_ | | 29 | |  |  |  |
| Observations | | 13152 | |  |  |  |
| Marginal R^2^ / Conditional R^2^ | | 0.048 / 0.259 | |  |  |  |

- **Folded wing length**

| **folded_wing** | | | | | | |
| --- | --- | --- | --- | --- | --- | --- |
| ***Predictors*** | ***Estimates*** | | ***std. Error*** | ***CI*** | | *p* |
| **(Intercept)** | 136.68 | | 0.28 | 136.13 – 137.22 | | **<0.001** |
| **Sex (female)** | 0.01 | | 0.03 | -0.05 – 0.06 | | 0.829 |
| **Age(adult)** | 0.30 | | 0.03 | 0.24 – 0.36 | | **<0.001** |
| **Julian day ^1** | -22.23 | | 4.49 | -31.02 – -13.44 | | **<0.001** |
| **Julian day ^2** | -21.01 | | 4.26 | -29.37 – -12.66 | | **<0.001** |
| **Julian day ^3** | 1.27 | | 3.90 | -6.37 – 8.91 | | 0.745 |
| **Year^1** | 2.69 | | 18.24 | -33.05 – 38.43 | | 0.883 |
| **Year^2** | -11.01 | | 19.22 | -48.68 – 26.67 | | 0.567 |
| **Year^3** | 3.84 | | 12.66 | -20.97 – 28.66 | | 0.762 |
| **Sex * Year^1** | -7.91 | | 3.64 | -15.04 – -0.78 | | **0.030** |
| **Sex * Year^2** | -5.30 | | 3.55 | -12.26 – 1.65 | | 0.135 |
| **Sex * Year^3** | 6.55 | | 3.97 | -1.23 – 14.34 | | 0.099 |
| **Julian day ^1 * Year^1** | 767.92 | | 591.94 | -392.25 – 1928.10 | | 0.195 |
| **Julian day ^2 * Year^1** | 2229.58 | | 611.43 | 1031.20 – 3427.96 | | **<0.001** |
| **Julian day ^3 * Year^1** | -417.95 | | 561.06 | -1517.61 – 681.71 | | 0.456 |
| **Julian day ^1 * Year^2** | 1193.56 | | 542.90 | 129.50 – 2257.62 | | **0.028** |
| **Julian day ^2 * Year^2** | 508.06 | | 568.41 | -606.00 – 1622.12 | | 0.371 |
| **Julian day ^3 * Year^2** | 381.29 | | 501.31 | -601.25 – 1363.83 | | 0.447 |
| **Julian day ^1 * Year^3** | -63.58 | | 632.81 | -1303.87 – 1176.71 | | 0.920 |
| **Julian day ^2 * Year^3** | -1694.78 | | 611.54 | -2893.39 – -496.18 | | **0.006** |
| **Julian day ^3 * Year^3** | 1018.72 | | 556.94 | -72.87 – 2110.31 | | 0.067 |
| **Random Effects** | | | | |  |  |
| σ^2^ | | 11.30 | | |  |  |
| τ_00_ _banding_area_ | | 1.91 | | |  |  |
| τ_00_ _season_ | | 0.73 | | |  |  |
| ICC | | 0.19 | | |  |  |
| N _banding_area_ | | 65 | | |  |  |
| N _season_ | | 39 | | |  |  |
| Observations | | 15098 | | |  |  |
| Marginal R^2^ / Conditional R^2^ | | 0.013 / 0.200 | | |  |  |

- **Body mass**

| **body_mass** | | | | | | |
| --- | --- | --- | --- | --- | --- | --- |
| ***Predictors*** | ***Estimates*** | | ***std. Error*** | ***CI*** | | ***p*** |
| **(Intercept)** | 103.69 | | 0.61 | 102.49 – 104.89 | | **<0.001** |
| **Sex (female)** | 0.61 | | 0.23 | 0.15 – 1.07 | | **0.009** |
| **Age(adult)** | 0.45 | | 0.10 | 0.25 – 0.65 | | **<0.001** |
| **Julian day ^1** | 237.54 | | 16.28 | 205.64 – 269.44 | | **<0.001** |
| **Julian day ^2** | -133.50 | | 15.86 | -164.58 – -102.43 | | **<0.001** |
| **Julian day ^3** | 27.85 | | 13.71 | 0.98 – 54.73 | | **0.042** |
| **Year^1** | 119.09 | | 40.90 | 38.92 – 199.26 | | **0.004** |
| **Year^2** | -147.48 | | 43.28 | -232.31 – -62.65 | | **0.001** |
| **Year^3** | -106.31 | | 35.82 | -176.52 – -36.11 | | **0.003** |
| **Recovery (Hunting recovery)** | -0.53 | | 0.24 | -1.01 – -0.05 | | **0.031** |
| **Sex * Recovery** | -0.46 | | 0.23 | -0.92 – -0.01 | | **0.047** |
| **Age * Julian day ^1** | 43.38 | | 14.08 | 15.79 – 70.98 | | **0.002** |
| **Age * Julian day ^2** | -65.63 | | 14.10 | -93.26 – -38.00 | | **<0.001** |
| **Age * Julian day ^3** | 86.91 | | 12.75 | 61.92 – 111.90 | | **<0.001** |
| **Julian day ^1 * Year^1** | 3810.00 | | 1762.22 | 356.12 – 7263.88 | | **0.031** |
| **Julian day ^2 * Year^1** | -1156.51 | | 1779.99 | -4645.22 – 2332.21 | | 0.516 |
| **Julian day ^3 * Year^1** | 6636.35 | | 1659.28 | 3384.21 – 9888.49 | | **<0.001** |
| **Julian day ^1 * Year^2** | 2905.20 | | 1642.67 | -314.38 – 6124.78 | | 0.077 |
| **Julian day ^2 * Year^2** | -473.19 | | 1673.46 | -3753.11 – 2806.73 | | 0.777 |
| **Julian day ^3 * Year^2** | 2913.73 | | 1520.42 | -66.25 – 5893.70 | | 0.055 |
| **Julian day ^1 * Year^3** | -1177.80 | | 1916.35 | -4933.77 – 2578.17 | | 0.539 |
| **Julian day ^2 * Year^3** | 4186.19 | | 1831.27 | 596.98 – 7775.41 | | **0.022** |
| **Julian day ^3 * Year^3** | -4179.33 | | 1714.56 | -7539.81 – -818.85 | | **0.015** |
| **Random Effects** | | | | |  |  |
| σ^2^ | | 101.17 | | |  |  |
| τ_00_ _banding_area_ | | 5.97 | | |  |  |
| τ_00_ _season_ | | 3.41 | | |  |  |
| ICC | | 0.08 | | |  |  |
| N _banding_area_ | | 65 | | |  |  |
| N _season_ | | 38 | | |  |  |
| Observations | | 15032 | | |  |  |
| Marginal R^2^ / Conditional R^2^ | | 0.074 / 0.153 | | |  |  |


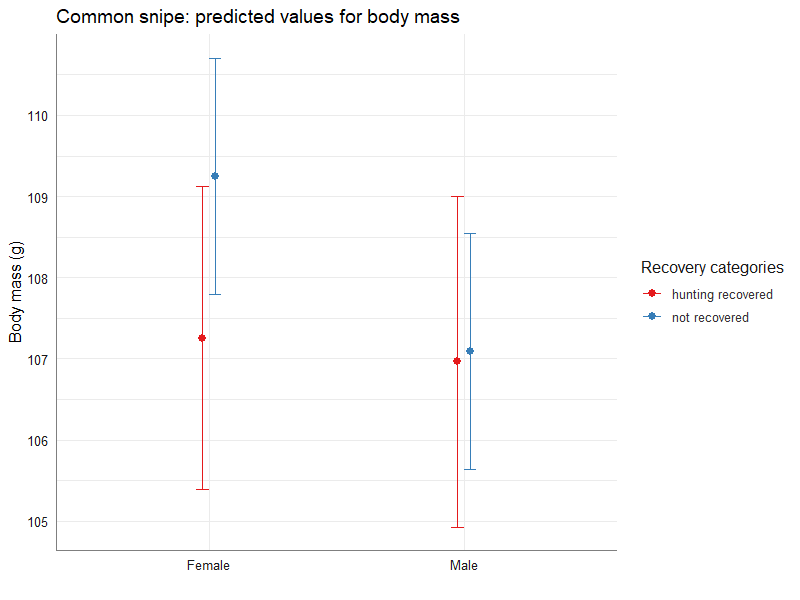


Figure B7: Predicted values of body mass as a function of Sex and Recovery category in Common Snipe (*Gallinago gallinago*).

- **Body condition index**

| **body_condition** | | | | | | |
| --- | --- | --- | --- | --- | --- | --- |
| ***Predictors*** | ***Estimates*** | | ***std. Error*** | ***CI*** | | ***p*** |
| **(Intercept)** | 103.24 | | 1.05 | 101.18 – 105.30 | | **<0.001** |
| **Sex (female)** | 0.99 | | 0.11 | 0.78 – 1.20 | | **<0.001** |
| **Age(adult)** | -0.35 | | 0.13 | -0.60 – -0.09 | | **0.008** |
| **Julian day ^1** | 284.93 | | 20.85 | 244.07 – 325.79 | | **<0.001** |
| **Julian day ^2** | -53.53 | | 20.34 | -93.39 – -13.66 | | **0.008** |
| **Julian day ^3** | 20.32 | | 17.52 | -14.02 – 54.65 | | 0.246 |
| **Year^1** | 137.80 | | 64.14 | 12.08 – 263.52 | | **0.032** |
| **Year^2** | -135.24 | | 67.98 | -268.49 – -2.00 | | **0.047** |
| **Year^3** | -145.78 | | 52.50 | -248.68 – -42.89 | | **0.005** |
| **Recovery (Hunting recovery)** | -0.88 | | 0.31 | -1.48 – -0.28 | | **0.004** |
| **Age * Julian day ^1** | 26.77 | | 17.90 | -8.32 – 61.86 | | 0.135 |
| **Age * Julian day ^2** | -41.05 | | 17.94 | -76.22 – -5.88 | | **0.022** |
| **Age * Julian day ^3** | 87.87 | | 16.22 | 56.08 – 119.65 | | **<0.001** |
| **Julian day ^1 * Year^1** | 1549.00 | | 2287.69 | -2934.80 – 6032.80 | | 0.498 |
| **Julian day ^2 * Year^1** | -7927.17 | | 2322.32 | -12478.84 – -3375.49 | | **0.001** |
| **Julian day ^3 * Year^1** | 7993.02 | | 2150.67 | 3777.78 – 12208.26 | | **<0.001** |
| **Julian day ^1 * Year^2** | -1131.78 | | 2131.24 | -5308.94 – 3045.37 | | 0.595 |
| **Julian day ^2 * Year^2** | -1968.87 | | 2170.37 | -6222.72 – 2284.97 | | 0.364 |
| **Julian day ^3 * Year^2** | 1719.40 | | 1964.29 | -2130.55 – 5569.34 | | 0.381 |
| **Julian day ^1 * Year^3** | -1365.45 | | 2465.55 | -6197.84 – 3466.93 | | 0.580 |
| **Julian day ^2 * Year^3** | 9617.48 | | 2360.43 | 4991.11 – 14243.85 | | **<0.001** |
| **Julian day ^3 * Year^3** | -7113.30 | | 2200.68 | -11426.55 – -2800.05 | | **0.001** |
| **Random Effects** | | | | |  |  |
| σ^2^ | | 162.31 | | |  |  |
| τ_00_ _banding_area_ | | 27.45 | | |  |  |
| τ_00_ _season_ | | 8.90 | | |  |  |
| ICC | | 0.18 | | |  |  |
| N _banding_area_ | | 65 | | |  |  |
| N _season_ | | 38 | | |  |  |
| Observations | | 14897 | | |  |  |
| Marginal R^2^ / Conditional R^2^ | | 0.048 / 0.222 | | |  |  |

- **Blackbird**
- **Folded wing length**

| **folded_wing** | | | | | | |
| --- | --- | --- | --- | --- | --- | --- |
| ***Predictors*** | ***Estimates*** | | ***std. Error*** | ***CI*** | | ***p*** |
| **(Intercept)** | 127.17 | | 0.24 | 126.71 – 127.64 | | **<0.001** |
| **Sex (female)** | -2.09 | | 0.01 | -2.12 – -2.07 | | **<0.001** |
| **Age(adult)** | 0.72 | | 0.01 | 0.69 – 0.74 | | **<0.001** |
| **Julian day ^1** | 115.07 | | 5.15 | 104.98 – 125.16 | | **<0.001** |
| **Julian day ^2** | -225.12 | | 5.11 | -235.14 – -215.10 | | **<0.001** |
| **Julian day ^3** | 26.90 | | 4.64 | 17.80 – 35.99 | | **<0.001** |
| **Year^1** | 225.68 | | 22.48 | 181.61 – 269.74 | | **<0.001** |
| **Year^2** | -8.12 | | 18.38 | -44.15 – 27.91 | | 0.659 |
| **Year^3** | -22.59 | | 17.23 | -56.36 – 11.17 | | 0.190 |
| **Recovery (Hunting recovery)** | 0.45 | | 0.19 | 0.08 – 0.82 | | **0.017** |
| **Sex * Age** | -0.35 | | 0.01 | -0.38 – -0.33 | | **<0.001** |
| **Sex * Julian day ^1** | -15.18 | | 4.06 | -23.14 – -7.22 | | **<0.001** |
| **Sex * Julian day ^2** | 24.56 | | 3.85 | 17.02 – 32.10 | | **<0.001** |
| **Sex * Julian day ^3** | -6.99 | | 3.81 | -14.47 – 0.48 | | 0.067 |
| **Sex * Year^1** | -26.48 | | 4.66 | -35.62 – -17.34 | | **<0.001** |
| **Sex * Year^2** | -0.23 | | 4.66 | -9.37 – 8.91 | | 0.960 |
| **Sex * Year^3** | -0.36 | | 4.50 | -9.19 – 8.47 | | 0.937 |
| **Age * Julian day ^1** | 54.34 | | 4.54 | 45.43 – 63.25 | | **<0.001** |
| **Age * Julian day ^2** | -68.41 | | 4.68 | -77.59 – -59.23 | | **<0.001** |
| **Age * Julian day ^3** | 41.58 | | 4.18 | 33.38 – 49.78 | | **<0.001** |
| **Age * Year^1** | 27.45 | | 5.65 | 16.38 – 38.52 | | **<0.001** |
| **Age * Year^2** | 6.92 | | 5.66 | -4.18 – 18.02 | | 0.222 |
| **Age * Year^3** | 11.46 | | 5.57 | 0.54 – 22.38 | | **0.040** |
| **Julian day ^1 * Year^1** | 2505.77 | | 1961.96 | -1339.60 – 6351.14 | | 0.202 |
| **Julian day ^2 * Year^1** | -12800.13 | | 1841.90 | -16410.20 – -9190.06 | | **<0.001** |
| **Julian day ^3 * Year^1** | -3581.12 | | 1828.15 | -7164.22 – 1.98 | | 0.050 |
| **Julian day ^1 * Year^2** | -3818.78 | | 1807.80 | -7361.99 – -275.56 | | **0.035** |
| **Julian day ^2 * Year^2** | -12656.92 | | 1763.41 | -16113.15 – -9200.69 | | **<0.001** |
| **Julian day ^3 * Year^2** | 3263.44 | | 1733.15 | -133.47 – 6660.34 | | 0.060 |
| **Julian day ^1 * Year^3** | 182.34 | | 1874.93 | -3492.45 – 3857.14 | | 0.923 |
| **Julian day ^2 * Year^3** | 5925.69 | | 1745.25 | 2505.06 – 9346.32 | | **0.001** |
| **Julian day ^3 * Year^3** | -9741.58 | | 1770.05 | -13210.81 – -6272.35 | | **<0.001** |
| **Random Effects** | | | | |  |  |
| σ^2^ | | 10.83 | | |  |  |
| τ_00_ _banding_area_ | | 1.68 | | |  |  |
| τ_00_ _season_ | | 0.16 | | |  |  |
| ICC | | 0.15 | | |  |  |
| N _banding_area_ | | 96 | | |  |  |
| N _season_ | | 66 | | |  |  |
| Observations | | 100087 | | |  |  |
| Marginal R^2^ / Conditional R^2^ | | 0.285 / 0.389 | | |  |  |

- **Body mass**

| **body_mass** | | | | | | |
| --- | --- | --- | --- | --- | --- | --- |
| ***Predictors*** | ***Estimates*** | | ***std. Error*** | ***CI*** | | ***p*** |
| **(Intercept)** | 91.55 | | 0.30 | 90.96 – 92.13 | | **<0.001** |
| **Sex (female)** | -1.08 | | 0.03 | -1.15 – -1.02 | | **<0.001** |
| **Age(adult)** | 0.42 | | 0.04 | 0.35 – 0.50 | | **<0.001** |
| **Julian day ^1** | 637.85 | | 13.34 | 611.71 – 663.99 | | **<0.001** |
| **Julian day ^2** | -646.68 | | 13.39 | -672.93 – -620.42 | | **<0.001** |
| **Julian day ^3** | -523.56 | | 12.35 | -547.77 – -499.34 | | **<0.001** |
| **Year^1** | -82.11 | | 54.28 | -188.49 – 24.27 | | 0.130 |
| **Year^2** | -192.68 | | 44.45 | -279.81 – -105.55 | | **<0.001** |
| **Year^3** | 115.65 | | 41.17 | 34.95 – 196.35 | | **0.005** |
| **Sex * Age** | -0.08 | | 0.03 | -0.13 – -0.02 | | **0.009** |
| **Sex * Julian day ^1** | 45.80 | | 10.04 | 26.13 – 65.48 | | **<0.001** |
| **Sex * Julian day ^2** | 116.36 | | 9.55 | 97.65 – 135.07 | | **<0.001** |
| **Sex * Julian day ^3** | 42.26 | | 9.55 | 23.54 – 60.97 | | **<0.001** |
| **Sex * Year^1** | -35.82 | | 12.92 | -61.14 – -10.50 | | **0.006** |
| **Sex * Year^2** | 12.73 | | 12.21 | -11.20 – 36.65 | | 0.297 |
| **Sex * Year^3** | -6.56 | | 10.94 | -27.99 – 14.88 | | 0.549 |
| **Age * Julian day ^1** | 36.58 | | 11.09 | 14.84 – 58.33 | | **0.001** |
| **Age * Julian day ^2** | -12.22 | | 11.39 | -34.55 – 10.11 | | 0.284 |
| **Age * Julian day ^3** | -93.84 | | 10.37 | -114.15 – -73.52 | | **<0.001** |
| **Age * Year^1** | -46.61 | | 17.30 | -80.51 – -12.71 | | **0.007** |
| **Age * Year^2** | 1.78 | | 16.38 | -30.33 – 33.90 | | 0.913 |
| **Age * Year^3** | -50.67 | | 14.41 | -78.91 – -22.43 | | **<0.001** |
| **Julian day ^1 * Year^1** | 7824.68 | | 5471.12 | -2898.53 – 18547.88 | | 0.153 |
| **Julian day ^2 * Year^1** | 27201.98 | | 5219.25 | 16972.44 – 37431.53 | | **<0.001** |
| **Julian day ^3 * Year^1** | -33650.94 | | 5170.83 | -43785.58 – -23516.31 | | **<0.001** |
| **Julian day ^1 * Year^2** | 36675.49 | | 4859.22 | 27151.59 – 46199.39 | | **<0.001** |
| **Julian day ^2 * Year^2** | -3641.74 | | 4778.03 | -13006.51 – 5723.03 | | 0.446 |
| **Julian day ^3 * Year^2** | -15556.87 | | 4707.21 | -24782.84 – -6330.90 | | **0.001** |
| **Julian day ^1 * Year^3** | 1392.33 | | 4674.28 | -7769.10 – 10553.76 | | 0.766 |
| **Julian day ^2 * Year^3** | 6854.37 | | 4336.78 | -1645.56 – 15354.30 | | 0.114 |
| **Julian day ^3 * Year^3** | -3550.65 | | 4419.37 | -12212.45 – 5111.15 | | 0.422 |
| **Random Effects** | | | | |  |  |
| σ^2^ | | 55.51 | | |  |  |
| τ_00_ _banding_area_ | | 6.32 | | |  |  |
| τ_00_ _season_ | | 0.90 | | |  |  |
| ICC | | 0.12 | | |  |  |
| N _banding_area_ | | 96 | | |  |  |
| N _season_ | | 66 | | |  |  |
| Observations | | 87796 | | |  |  |
| Marginal R^2^ / Conditional R^2^ | | 0.135 / 0.234 | | |  |  |

- **Body condition index**

| **body_condition** | | | | | | |
| --- | --- | --- | --- | --- | --- | --- |
| ***Predictors*** | ***Estimates*** | | ***std. Error*** | ***CI*** | | ***p*** |
| **(Intercept)** | 93.25 | | 0.30 | 92.66 – 93.84 | | **<0.001** |
| **Sex (female)** | 3.09 | | 0.03 | 3.02 – 3.15 | | **<0.001** |
| **Age(adult)** | -0.86 | | 0.05 | -0.96 – -0.77 | | **<0.001** |
| **Julian day ^1** | 386.89 | | 16.49 | 354.56 – 419.22 | | **<0.001** |
| **Julian day ^2** | -198.31 | | 16.56 | -230.77 – -165.84 | | **<0.001** |
| **Julian day ^3** | -571.40 | | 15.17 | -601.14 – -541.67 | | **<0.001** |
| **Year^1** | -490.83 | | 64.99 | -618.22 – -363.45 | | **<0.001** |
| **Year^2** | -255.19 | | 53.27 | -359.60 – -150.77 | | **<0.001** |
| **Year^3** | 176.19 | | 49.47 | 79.23 – 273.15 | | **<0.001** |
| **Sex (female) * Age** | 0.59 | | 0.04 | 0.52 – 0.66 | | **<0.001** |
| **Sex * Julian day ^1** | 94.45 | | 12.24 | 70.47 – 118.44 | | **<0.001** |
| **Sex * Julian day ^2** | 70.29 | | 11.44 | 47.87 – 92.72 | | **<0.001** |
| **Sex * Julian day ^3** | 36.00 | | 11.62 | 13.22 – 58.77 | | **0.002** |
| **Age * Julian day ^1** | -95.14 | | 13.84 | -122.26 – -68.01 | | **<0.001** |
| **Age * Julian day ^2** | 117.39 | | 14.28 | 89.40 – 145.39 | | **<0.001** |
| **Age * Julian day ^3** | -186.97 | | 12.78 | -212.02 – -161.92 | | **<0.001** |
| **Age * Year^1** | -105.87 | | 21.26 | -147.54 – -64.20 | | **<0.001** |
| **Age * Year^2** | 4.70 | | 19.98 | -34.46 – 43.86 | | 0.814 |
| **Age * Year^3** | -64.85 | | 17.77 | -99.67 – -30.03 | | **<0.001** |
| **Julian day ^1 * Year^1** | 6380.80 | | 6670.79 | -6693.71 – 19455.32 | | 0.339 |
| **Julian day ^2 * Year^1** | 51385.15 | | 6348.41 | 38942.50 – 63827.80 | | **<0.001** |
| **Julian day ^3 * Year^1** | -19397.38 | | 6295.18 | -31735.70 – -7059.05 | | **0.002** |
| **Julian day ^1 * Year^2** | 43362.92 | | 5906.01 | 31787.35 – 54938.49 | | **<0.001** |
| **Julian day ^2 * Year^2** | 23038.29 | | 5790.77 | 11688.58 – 34387.99 | | **<0.001** |
| **Julian day ^3 * Year^2** | -29140.85 | | 5701.25 | -40315.10 – -17966.60 | | **<0.001** |
| **Julian day ^1 * Year^3** | -469.83 | | 5695.22 | -11632.25 – 10692.60 | | 0.934 |
| **Julian day ^2 * Year^3** | -4497.32 | | 5276.27 | -14838.61 – 5843.97 | | 0.394 |
| **Julian day ^3 * Year^3** | 16023.03 | | 5373.45 | 5491.26 – 26554.81 | | **0.003** |
| **Random Effects** | | | | |  |  |
| σ^2^ | | 79.67 | | |  |  |
| τ_00_ _banding_area_ | | 5.49 | | |  |  |
| τ_00_ _season_ | | 1.28 | | |  |  |
| ICC | | 0.08 | | |  |  |
| N _banding_area_ | | 96 | | |  |  |
| N _season_ | | 66 | | |  |  |
| Observations | | 81718 | | |  |  |
| Marginal R^2^ / Conditional R^2^ | | 0.146 / 0.213 | | |  |  |
